# Advancing Selective Extraction: A Novel Approach for Scandium, Thorium, and Uranium Ion Capture

**DOI:** 10.1002/smsc.202400171

**Published:** 2024-08-28

**Authors:** Iryna Protsak, Martin Stockhausen, Aaron Brewer, Martin Owton, Thilo Hofmann, Freddy Kleitz

**Affiliations:** ^1^ Department of Functional Materials and Catalysis University of Vienna Währinger Straße 42 1090 Vienna Austria; ^2^ Department for Environmental Geosciences, Centre for Microbiology and Environmental Systems Science University of Vienna Josef‐Holaubek‐Platz 2 1190 Vienna Austria; ^3^ Independent Scholar Surrey UK

**Keywords:** mesoporous sorbent, scandium, selective extraction, thorium, uranium

## Abstract

The potential use of thorium (Th) and uranium (U) as nuclear fuels underscores the importance of developing materials for their sustainable recovery. The production of Th and U requires the separation of these elements from rare‐earth elements (REEs) as they often coexist in various feedstocks. Equally crucial is efficiently isolating scandium (Sc) from REEs, considering its high‐value status and pivotal role in advanced alloy technologies. This study introduces a new selective ligand‐functionalized silica sorbent for extracting Sc, other REEs, Th, and U from solutions with varying pH and elemental compositions. The functionalized sorbent exhibits exceptional selectivity for Sc ions at pH 4 across solutions containing 3–20 elements. It also shows excellent selectivity for Th at pH 2 in 18‐ and 20‐element solutions and substantial selectivity for U in 18‐ and 20‐element solutions at pH 4. Additionally, it efficiently adsorbs neodymium (Nd), dysprosium (Dy), and lanthanum (La) in Sc‐free solutions with a given preference for Nd. The ligand‐functionalized sorbent successfully undergoes ten cycles of reuse which along with its enhanced recovery performance toward targeted elements highlights its industrial application potential.

## Graphical abstract

This study introduces an advanced functionalized mesoporous silica sorbent that demonstrates effectiveness in selectively extracting Sc, Th, and U from complex multi‐element solutions containing REEs and competitive elements. It offers a dual solution: targeted extraction of Th, U, and Sc, and their separation from REEs, addressing a major challenge in critical materials recovery and separation.

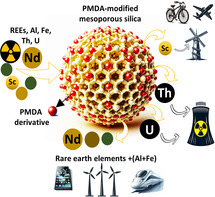

## 1 Introduction

The escalating global demand for energy, driven by globalization and rapid population growth, is turning attention toward carbon‐free and environmentally sustainable energy sources. This shift has spurred the expansion of infrastructures for low‐emission technologies, such as nuclear power plants that utilize actinides, and wind turbines, which rely heavily on rare‐earth elements (REEs) for permanent magnets. Concurrently, there is a growing demand for a broader range of REEs, including scandium (Sc), which has been recognized as a critical material by the European Commission^[^ ^1^ ^]^ and the US government in North America.^[^ ^2^ ^]^ Although not used in wind turbines, scandium's unique properties are invaluable in other sustainable technologies.^[^ ^3^ ^]^ For example, it enhances the efficiency of solid oxide fuel cells,^[^ ^4^ ^]^ offering a cleaner alternative to traditional combustion‐based power generation, and it is critical in developing lightweight, high‐strength aluminum alloys,^[^ ^4^, ^5^, ^6^, ^7^, ^8^ ^]^ vital for aerospace and fuel‐efficient vehicles, thus contributing to energy efficiency and emissions reduction. Despite its advantages, isolating Sc from parent ores is cost‐intensive, with low concentrations and high energy requirements for extraction and processing.^[^ ^5^, ^7^ ^]^ Consequently, exploring alternative, sustainable methods for the separation of Sc from REE solutions becomes a critical necessity.

In the realm of actinides, thorium (^232^Th (IV)) stands out for its potential usage in nuclear energy and inherent safety features.^[^ ^9^ ^]^ Its applications extend to catalysts,^[^ ^10^ ^]^ refractories,^[^ ^11^ ^]^ sensors,^[^ ^12^ ^]^ and solid electrolytes.^[^ ^13^ ^]^ Uranium, a key radionuclide,^[^ ^14^ ^]^ powers commercial nuclear reactors.^[^ ^15^, ^16^ ^]^ Considering that uranium (U) and thorium (Th) often coexist with lanthanides in rare‐earth deposits due to lattice substitution, and they are found alongside common metals like iron (Fe) and aluminum (Al) in natural deposits, their efficient separation from REE solutions is crucial.^[^ ^17^, ^18^, ^19^, ^20^, ^21^ ^]^ Additionally, the need to distinguish and separate Th and U from one another is equally critical, considering their overlapping presence in these complex mixtures. Therefore, developing highly efficient methods for the separation and selective recovery of thorium, uranium, and scandium from diverse REE solutions is of urgent importance.

Solid‐phase extraction (SPE) stands as a comprehensive category of techniques for recovering metals. It utilizes a solid material to selectively capture a specific component from an aqueous feedstock (like a mineral leachate) through adsorption. The purified aqueous metal is usually precipitated and roasted, resulting in a marketable solid product such as a metal oxide. SPE presents advantages, especially in terms of environmental impact, as these systems typically need minimal or no hazardous organic solvents for synthesis or use, differing from traditional liquid−liquid extraction approaches.^[^ ^6^ ^]^ A broad range of solid‐phase adsorbents, including nanoporous silicas,^[^ ^21^, ^22^ ^]^ carbon‐based materials,^[^ ^23^, ^24^, ^25^, ^26^ ^]^ covalent organic frameworks (COFs),^[^ ^5^, ^27^ ^]^ metal–organic frameworks,^[^ ^28^, ^29^ ^]^ and clays,^[^ ^30^ ^]^ among others, have been investigated for the recovery and separation of these elements. However, many of these materials face the significant challenge of inadequate selectivity, which is essential for the efficient recovery of specific ions. Enhanced selectivity in these materials can be achieved by incorporating specific functional groups within their structures and surfaces, often through targeted modifications. In materials such as COFs, clays, or carbon‐based materials, the functionalization process is limited by the lack of specific surface groups needed for further modifications. This deficiency necessitates additional chemical functionalization steps, which often incur extra expenses and are time‐consuming. In contrast, silica materials offer an excellent platform for straightforward modification due to the presence of silanol groups, which enable the grafting of diverse functionalities through coupling reactions with organosilanes.^[^ ^31^, ^32^ ^]^ Ordered mesoporous silica, particularly of the SBA‐15‐type, stands out as an exceptional candidate for such functionalization. It features a range of silanol groups, a high surface area, and relatively large pores.^[^ ^33^ ^]^ The uniform and well‐defined mesopore channels in SBA‐15 ensure better accessibility of active sites, which is crucial in catalysis and adsorption processes. Moreover, the morphology and size of the particles can be tuned,^[^ ^34^ ^]^ and the material can be shaped into monolithic structures, which is essential for industrial‐scale applications of future sorbents.^[^ ^35^, ^36^ ^]^ Hence, the discovery of ligands with superior selectivity becomes pivotal in enhancing the extraction performance of such silica materials. Pyromellitic dianhydride (PMDA), as a reactive anhydride, provides a straightforward approach for covalently attaching to amine‐functionalized silica through a ring‐opening approach.^[^ ^37^ ^]^ To the best of our knowledge, this specific anhydride has not been previously utilized in the context of sorbent preparation for the extraction of metal ions. Its interaction with amine‐functionalized silica facilitates the grafting of carboxyl groups onto the surface and within the pores, providing the sorbent with two active sites for the adsorption of metal ions: the carbonyl groups and carboxyl groups. Carbonyl groups participate in the chelation of metal ions, depending on the atomic radius of the metal ion, while carboxyl groups can coordinate with metal ions or engage in ionic interactions, influenced by both the pH of the solution and the atomic radius of the metal ion. This dual mechanism ensures the desired selectivity in adsorption. The present study will demonstrate that the ligand‐functionalized silica sorbent can effectively be employed for the selective extraction of Sc, other REEs, and radionuclides like Th and U from diverse multi‐element solutions. These solutions varied in the number of REEs and included key competitive elements, such as Al and Fe, across different pH conditions. Exhibiting exceptional selectivity and recyclability, the sorbent furthermore proves its promising potential for industrial applications.

## 2 Results and Discussion

**Figure 1:**
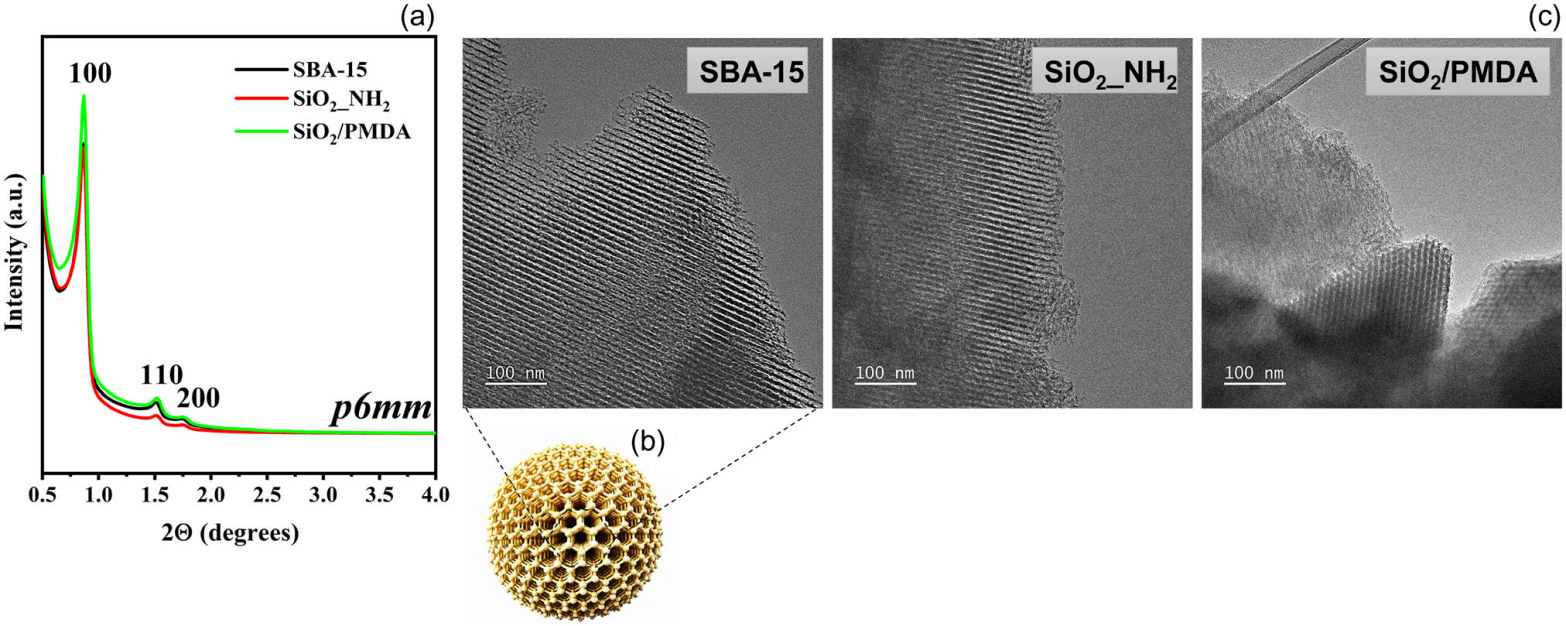
a) Low‐angle powder XRD patterns of the synthesized silica‐based materials. b) Spherical particle model which portrays an ordered mesostructure consisting of a symmetrical arrangement of mesoporous channels in SBA‐15. c) TEM images of SBA‐15, SBA_NH_2_, and SiO_2_/PMDA. The scale bar corresponds to 100 nm.

In this study, the SBA‐15 was synthesized using established protocols that facilitate the creation of silica materials with customizable properties and high quality.^[^ ^34^ ^]^ The resulting SBA‐15 was then functionalized with PMDA via a straightforward two‐step process, as detailed in the Supporting Information (Scheme S1). The corresponding X‐ray diffractograms of SBA‐15 and ligand‐functionalized silica revealed three well‐resolved diffraction peaks indexed as 100, 110, and 200 reflections, characteristic of a 2D hexagonal mesostructure with a pore symmetry of *p6mm* (**Figure** **1**a).^[^ ^33^ ^]^ The diffraction patterns of SiO_2_/PMDA align closely with those of the SBA‐15 precursor, confirming the retention of an ordered mesoporous structure after surface functionalization. Figure 1c and Figure S1 display transmission electron microscopy (TEM) images of all materials, emphasizing a periodic alignment of channel‐like mesopores (and spherical particle model portrays an ordered mesostructure, consisting of a symmetrical arrangement of mesoporous channels in SBA‐15 (Figure 1b)). The TEM images, particularly those in Figure S1, distinctly reveal the hexagonal geometry of these mesopores, which aligns with and supports the findings from the low‐angle X‐ray diffraction (XRD) data.

**Figure 2:**
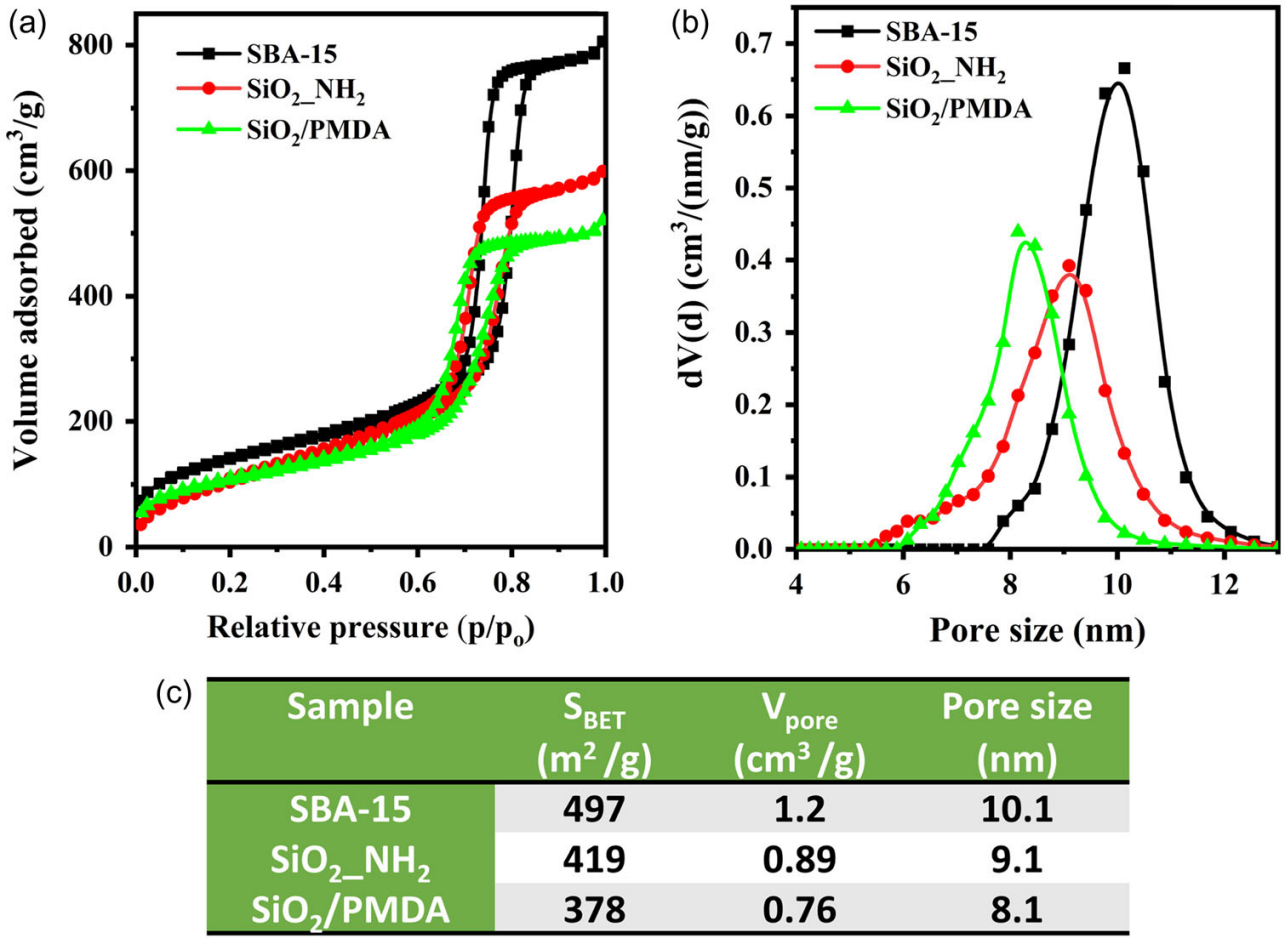
a) N_2_ adsorption–desorption isotherms at −196 °C for the synthesized materials and b) respective pore size distributions calculated from the equilibrium branch using the NLDFT method (silica with cylindrical pore model). c) Table showing the physicochemical parameters derived from N_2_ physisorption measurements at −196 °C.

The N_2_ physisorption isotherms are presented in **Figure** **2**a, and the physicochemical parameters (porosity and structural parameters) derived from the N_2_ sorption analysis are compiled in Figure 2b,c. All samples exhibited a characteristic type IV isotherm. A steep capillary condensation step and a hysteresis loop (type H1 adsorption–desorption hysteresis) were observed in the relative pressure range (*P*/*P*_o_) of 0.6–0.8 (Figure 2a). NLDFT pore size distributions (Figure 2b) were relatively narrow for the modified materials, which suggests that mesoporous silica preserved well‐defined and uniform mesopores after functionalization. According to the non‐local density functional theory (NLDFT) pore size distributions, SBA‐15 possessed a pore size of 10.1 nm (Figure 2c). After the initial modification step, the pore size of SBA‐15_NH_2_ decreased to 9.1 nm, and following the second modification step, it further reduced to 8.1 nm for SiO_2_/PMDA. Likewise, the specific surface area and pore volume decreased after functionalization, transitioning from 497 to 419 m^2^ g^−1^ for aminated silica and from 497 to 378 m^2^ g^−1^ for silica modified with PMDA, respectively. The initial reduction in surface area aligns with the introduction of 3‐(aminopropyl)triethoxysilane (APTES), and the subsequent reduction in surface area aligns with PMDA attachment. The solid‐state ^29^Si cross polarization/magic angle spinning (CP/MAS) NMR spectrum of bare silica (Figure S2a) shows shifts centered at −91, −101, and −110 ppm, corresponding to Q^2^, Q^3^, and Q^4^ species.^[^ ^38^, ^39^ ^]^ These shifts represent geminal silanols, vicinal silanols, and fully condensed silica network species, respectively. After modification with PMDA, the silica exhibits T^2^ and T^3^ signals (Figure S2a), confirming the successful chemical modification of the silica surface.^[^ ^40^, ^41^, ^42^ ^]^ In the solid‐state ^13^C CP/MAS NMR spectrum of SiO_2__NH_2_, Figure S2b, three resonance signals were detected at 42, 24, and 8 ppm, ascribed to the carbons of the silane propyl chain. Furthermore, after SiO_2__NH_2_ modification with PMDA, additional peaks emerged at 132 ppm and 169 ppm, confirming the presence of an aromatic ring and carbonyl groups, respectively, at the silica surface and within the pores.

Surface functionalization of SBA‐15 was confirmed using infrared (IR) spectroscopy, as shown in Figure S3. The IR spectra for both SiO_2__NH_2_ and SiO_2_/PMDA samples exhibit absorption bands in the 2962–2871 cm^−1^ region, corresponding to C—H stretching vibrations. These bands confirm the presence of propyl groups.^[^ ^43^, ^44^ ^]^ The absorption band ranging from 1717 to 1627 cm^−1^ is indicative of the C=O stretching vibration, characteristic of both carboxylic and amide groups. Additionally, the IR spectrum of the PMDA‐modified silica reveals stretching vibrations of the C=C benzene ring, characteristic of the grafted PMDA moiety, in the range of 1600–1500 cm^−1^. An in‐plane N—H bending is observed at 1549 cm^−1^ for the PMDA‐modified silica. A pronounced asymmetric stretching vibration at 1044 cm^−1^, common to all samples, signifies the Si—O—Si asymmetric stretch within the SiO_4_ tetrahedra. Additionally, the IR spectra exhibit symmetric stretching vibrations of Si—O—Si at 797 cm^−1^ and out‐of‐plane deformations at 438 cm^−1^, which are characteristic signals of silica‐based materials.^[^ ^45^ ^]^ Thermogravimetric analysis, Figure S4a,b, reveals a weight loss of 9.3 wt% for SiO_2__NH_2_ and 22.8 wt% for SiO_2_/PMDA attributed to the thermal decomposition of the organic moieties on the functionalized silica between 150 and 800 °C. Furthermore, the attachment of PMDA is also confirmed by elemental analysis, revealing the presence of carbon at the PMDA‐modified silica surface which is higher than the carbon content of the aminated silica (Figure S4b).

Investigating the structure of the developed PMDA‐modified silica sorbent was crucial, as it reveals potential adsorption centers and their potential reactivity. Also, understanding this structure is key to delving deeper into the mechanisms of adsorption between the PMDA‐modified silica and the metal ions studied in this work. The reactive anhydride has the potential to interact with aminated silica through two distinct reaction pathways: either a mono‐sided reaction (as depicted in Scheme S2a) or a bis‐sided reaction (illustrated in Scheme S2b). This leads to the formation of two diverse PMDA derivatives anchored within the silica matrix. In the mono‐sided reaction, the subsequent washing protocol may initiate the opening of the second anhydride moiety. This process potentially results in the emergence of two additional carboxylic groups within this moiety, thereby becoming available for metal ion adsorption. Conversely, a bis‐sided reaction with SiO_2__NH_2_ (Scheme S2b) could result in the conversion of the carboxyl group into amide functionality, consequently restricting the rotational freedom of the associated carbonyl group. However, in the mono‐sided scenario, the carbonyl groups maintain greater rotational flexibility. Thus, it can be anticipated that the structure resulting from a mono‐sided reaction will exhibit greater reactivity due to its configuration. However, even though the structure formed by a bis‐sided reaction contains two amine functionalities that might limit the free rotation of the corresponding carbonyl groups, it still possesses two additional free carboxylic groups within this moiety. These groups retain their capacity to interact with metal ions, thus maintaining a significant level of reactivity.

**Figure 3:**
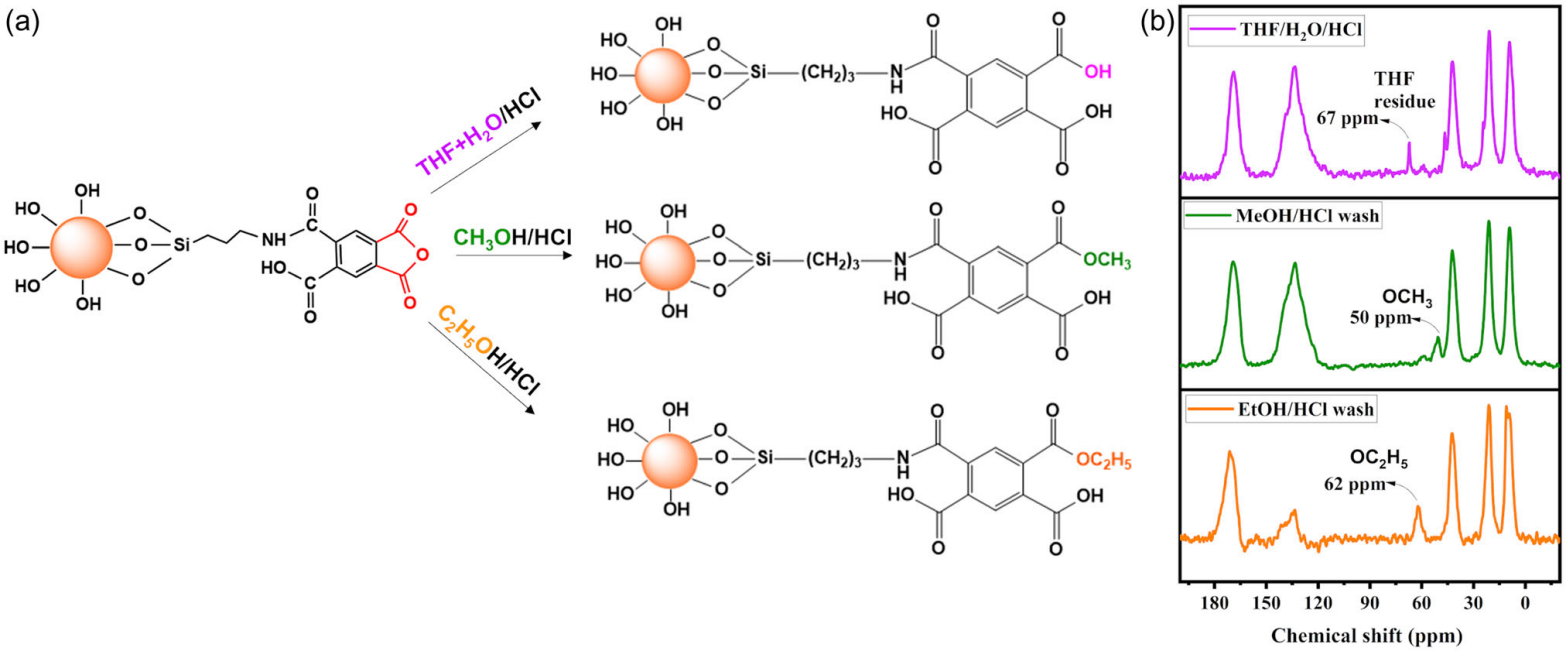
a) Schematic representation of potential SiO_2_/PMDA structure formations using different washing protocols. b) ^13^C CP/MAS NMR spectra of SiO_2_/PMDA samples subjected to three distinct washing procedures.

The structure of SiO_2_/PMDA was elucidated using an approach that involved washing the newly synthesized sample with different solvents (**Figure** **3**a): nucleophilic ones like methanol and ethanol, as well as the non‐nucleophilic solvent THF. The rationale for using nucleophilic solvents was to react with any unreacted anhydride on the silica surface, leading to the formation of new functional groups within the sorbent's structure (Figure 3a). These newly formed groups would remain intact even after a subsequent washing step with an organic solvent. In contrast, the use of a non‐nucleophilic solvent‐like THF aimed to determine whether the modified silica's structure could inherently retain solvent molecules. If retained, these solvent molecules could then be easily removed in a secondary washing step.

With a postwashing at the first step, solid‐state NMR analysis (Figure 3b, with lines from top to bottom) showed a residual THF signal post‐THF washing (violet line), formation of a methoxy group post‐methanol washing (green line), and an ethoxy group post‐ethanol washing (orange line). Further washing with a solvent–water mix aimed to remove any ethoxy, methoxy, or THF residues. This step was crucial to confirm whether these groups were integrally bonded to the grafted ligand.

**Figure 4:**
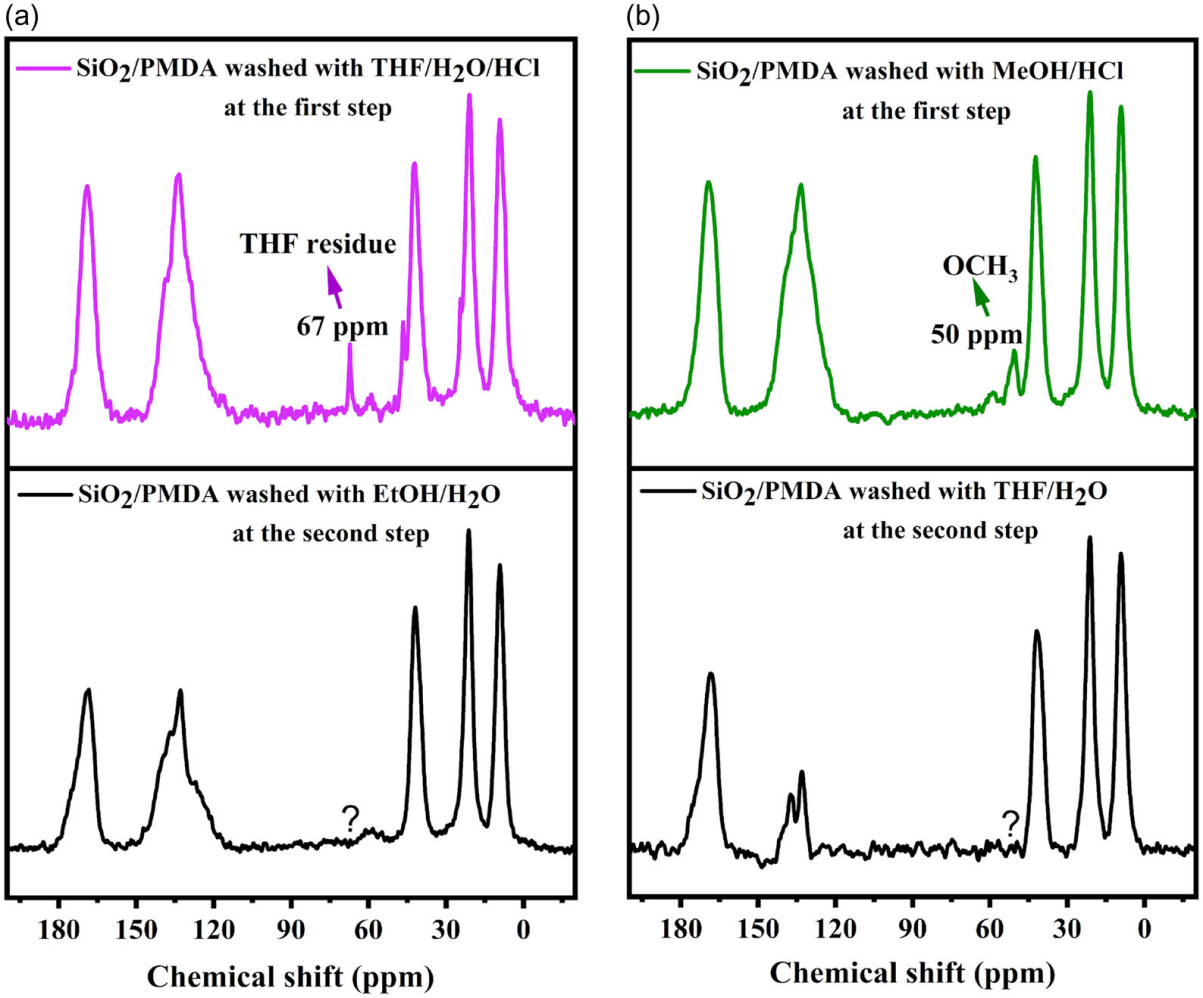
a) ^13^C CP/MAS NMR spectra of SiO_2_/PMDA initially washed with THF//HCl/water followed by a second washing with a different solvent/water mixture. b) ^13^C CP/MAS NMR spectra of SiO_2_/PMDA initially washed with MeOH/HCl followed by a secondary washing with a different solvent/water mixture.

The solid‐state NMR spectra after the second post‐washing (**Figure** **4**a,b) showed the disappearance of the THF peak in the ethanol/water‐washed sample (Figure 4a, violet line to black line) and the disappearance of the methoxy group in the THF/water washed sample (Figure 4b, green line to black line). This implies that the ethoxy and methoxy groups were only solvent residues, not reacting with any “unreacted” anhydride, suggesting the absence of such anhydride groups at the silica surface, and thus confirming the formation of the “b” structure as shown in Scheme S2.

The confirmation of the “b” structure, which is shown in Scheme S2, indicates that the aminated silica used as a precursor for subsequent reactions with PMDA features closely spaced amine groups and this finding is important for the targeted synthesis of SBA‐15 in future studies. By manipulating the synthesis temperature, it is possible to adjust the density and proximity of silanol groups, which, in turn, influences the extent of amine functionalization. Specifically, when silanol groups are sufficiently spaced which will lead to sufficiently spaced amine groups in the second modification step, pyromellitic dianhydride can react with an amine predominantly from one side. The unreacted side of the grafted anhydride could subsequently be activated with a targeted nucleophilic solvent, potentially leading to the formation of functional groups with enhanced adsorption activity which represents a promising avenue for future research.

The aminated silica, prepared for further reaction with pyromellitic dianhydride, is omitted from extraction tests due to its positive charge under acidic conditions,^[^ ^46^, ^47^ ^]^ limiting its efficacy for extracting positively charged ions. Analysis of structural and textural changes, detailed earlier, focused on the modification effects on surface area, pore size, and pore volume. Modification by APTES and then PMDA slightly altered the textural properties (see Figure 1) without significantly affecting the silica's inherent mesostructure. The scandium adsorption isotherms measured for SBA‐15 and SiO_2_/PMDA, as depicted in Figure S5, exhibit an initial steep rise, indicative of a strong affinity between the sorbent framework and Sc ions, before reaching a gradual plateau. However, the adsorption capacity of SiO_2_/PMDA‐modified silica is higher compared to that of SBA‐15 silica which can be attributed to the variation in reactive sites on each. Zeta potential measurements, as shown in Figure S6, indicate that the surface of modified silica at pH 4 possesses a strongly negative charge, suggesting an abundance of deprotonated carboxyl groups. These groups have a well‐documented affinity for REEs, as evidenced by numerous studies.^[^ ^48^, ^49^, ^50^ ^]^ Additionally, the interaction of Sc ions with SiO_2_/PMDA may be facilitated through chelation by carbonyl groups, further increasing the number of effective sites for ion adsorption. However, the feasibility of Sc ion chelation by carbonyl groups depends significantly on the ionic radius of Sc and the size of the chelate, a topic we explore later in the context of adsorption in the presence of larger ions. Bare silica, on the other hand, exhibits significantly less negative charge at pH 4, as depicted in Figure S6, indicating a reduced presence of negatively charged groups on the surface. The isotherm data for both materials were analyzed using the Langmuir and Freundlich models, with the Langmuir isotherm showing a better fit for both (*R* ^2^ = 0.97 compared to 0.95 and 0.94 for the Freundlich model, respectively, as shown in Table S1). The consistency of the experimental data with the Langmuir isotherm model implies that the sorption sites within the structures of SBA‐15 and PMDA‐modified silica are finite and homogeneous and suggests monolayer sorption.^[^ ^22^ ^]^

As depicted in Figure S7, the adsorption kinetics of Sc ions on PMDA‐modified silica show a rapid increase, reaching equilibrium within 134 min. This rate is consistent with other studies focusing on Sc and REEs recovery.^[^ ^5^, ^48^, ^51^, ^52^ ^]^ Also, modified silica shows a higher adsorption capacity than SBA‐15, indicating a stronger affinity toward Sc ions. Bare silica, in comparison, exhibits slower kinetics, reaching equilibrium in 480 min. Both kinetic analyses for the modified silica and bare SBA‐15 closely follow the pseudo‐second‐order kinetic model (PSO), evidenced by *R* ^2^ values of 0.99 for SiO_2_/PMDA and 0.98 for SBA‐15 (Figure S7, Table S2). The PSO model is often associated with chemisorption, which predominantly drives the adsorption process.^[^ ^22^ ^]^ The observation supports the likelihood of Sc ions forming coordinative bonds with the adsorption centers of both materials. The observed faster kinetics with modified silica, compared to SBA‐15, could be attributed to the chemistry of the carboxylate group (COO–). As demonstrated in other studies,^[^ ^48^, ^49^, ^50^ ^]^ the carboxylate group exhibits a strong affinity for adsorbing metal ions, particularly in its deprotonated form.^[^ ^53^ ^]^ In a carboxylate ion, the negative charge is delocalized over two oxygen atoms due to resonance, effectively spreading the electron density. This delocalization enables both oxygen atoms to share the negative charge equally which may enhance their capacity to coordinate effectively with Sc ions.

**Figure 5:**
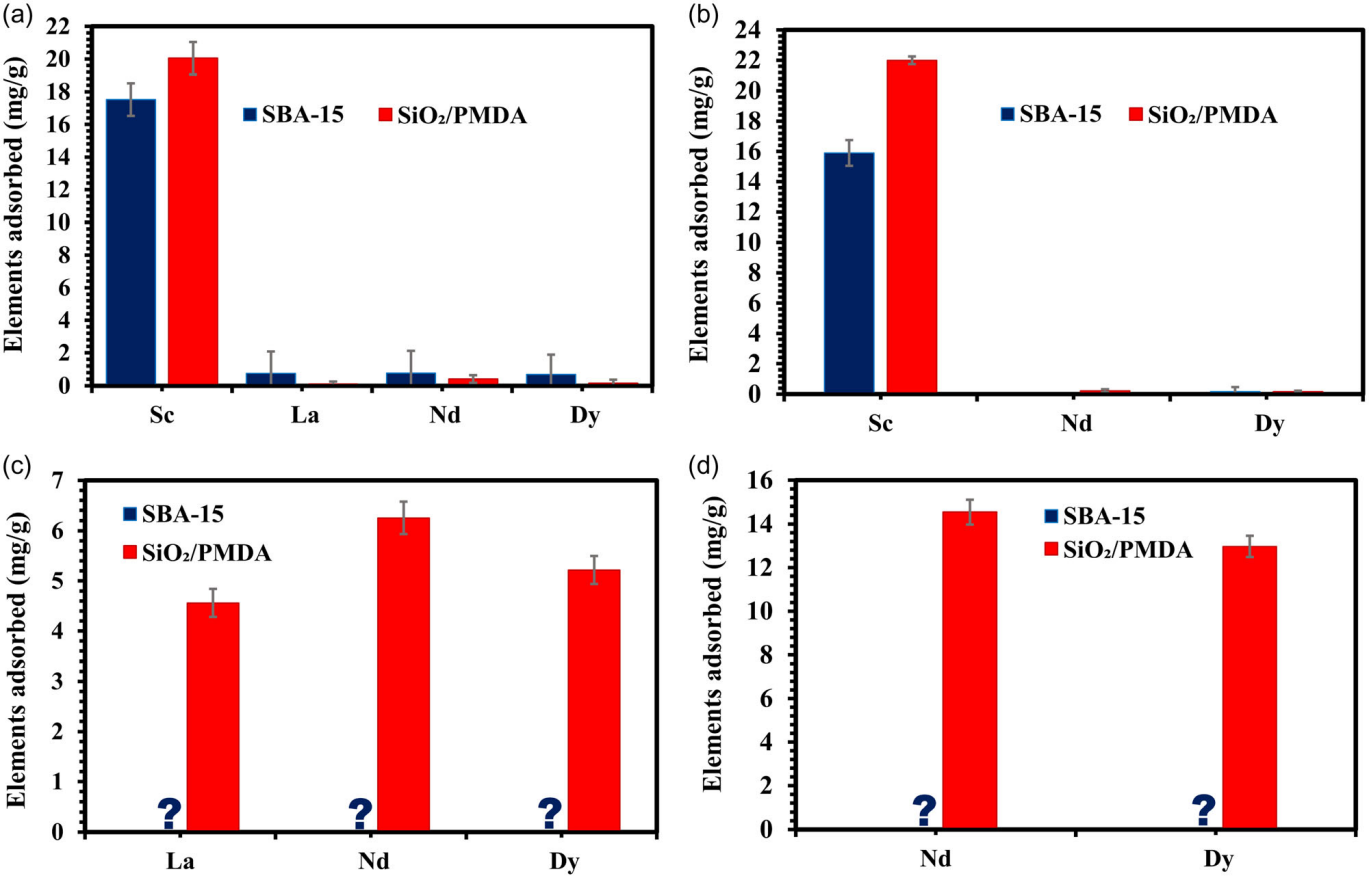
a) Adsorption of a 4‐REE solution (including Sc) by SiO_2_/PMDA and bare SBA‐15. b) Adsorption of a 3‐REE solution (including Sc) by SiO_2_/PMDA and SBA‐15. c) Adsorption of a 3‐REE solution (excluding Sc) by SiO_2_/PMDA and SBA‐15. d) Adsorption of a 2‐REE solution (excluding Sc) by SiO_2_/PMDA and SBA‐15. All tests were conducted at a pH of 4, with each element in the solution having a concentration of 34 mg L^−1^. Error bars indicate the standard deviation of triplicate measurements. The question mark on the graphs (c,d) is used to highlight the lack of affinity of SBA‐15 toward REEs.

The adsorption behavior of Sc in multi‐element REE solutions was studied to assess the selectivity of the newly developed sorbent, SiO_2_/PMDA, and SBA‐15 in comparison (**Figure** **5**).

As illustrated in Figure 5a, in a model 4‐REE solution at pH 4, both modified silica and bare SBA‐15 exhibit a strong preference for Sc ions, exclusively adsorbing them. This pattern continues in the 3‐REE solution (Figure 5b), where, again, both materials adsorb only Sc ions. The exceptional selectivity toward Sc ions exhibited by both materials can be attributed to Sc's small ionic radius compared to other REEs. Additionally, the kinetics of adsorption on the modified silica surface may also contribute to this selectivity. Notably, while equilibrium is reached for Nd adsorption after 180 min (as demonstrated in Figure S8, Table S3), the modified silica achieves saturation with Sc ions within 134 min (as shown in Figure S7).

In the 3‐REE elements solution without scandium (Figure 5c), SBA‐15 shows no adsorption for any of the present ions, whereas the modified silica demonstrates significant adsorption of La, Nd, and Dy ions with the given preference to Nd. In the 2‐REE solution (Figure 5d) containing only Nd and Dy ions (and no Sc ions), the modified silica continues to show substantial adsorption for these two ions, unlike SBA‐15, which does not adsorb either. Thus, the PMDA‐modified silica exhibits a dynamic, switchable adsorption behavior, which is responsive to the varying ionic compositions present in the solution. This adaptability in adsorption demonstrates the material's capacity to selectively target specific ions based on their unique chemical properties, thereby enabling tailored adsorption processes dependent on the ion‐specific environment.

The robust affinity of functionalized silica toward other REEs is further evidenced through a single‐element adsorption test with Nd. As shown in Figure S9, the adsorption profile for Nd on functionalized silica features an initial rapid increase, demonstrating a strong affinity between the sorbent and Nd ions, followed by a leveling off at a plateau. In contrast, SBA‐15 exhibits no adsorption of Nd, as evidenced by a flat line in the adsorption profile (Figure S9). This observation aligns with the REE selectivity tests described earlier, reinforcing the limited adsorption capabilities of bare silica compared to the more versatile and effective performance of the modified silica. The adsorption of other REEs by modified silica is enhanced by the presence of resonance‐stabilized, electron‐rich ionized COOH groups^[^ ^48^, ^49^, ^50^ ^]^ and carbonyl groups^[^ ^47^ ^]^ within the sorbent structure, which collectively attract both small and larger ions broadening the range of REEs effectively adsorbed. This, in turn, highlights the significance of modifying silica with organic ligands that endow the surface with a variety of functional groups which are chemically distinct from those on bare silica, and offer enhanced and more versatile adsorption capabilities.^[^ ^47^, ^54^, ^55^, ^56^, ^57^ ^56]^

**Figure 6:**
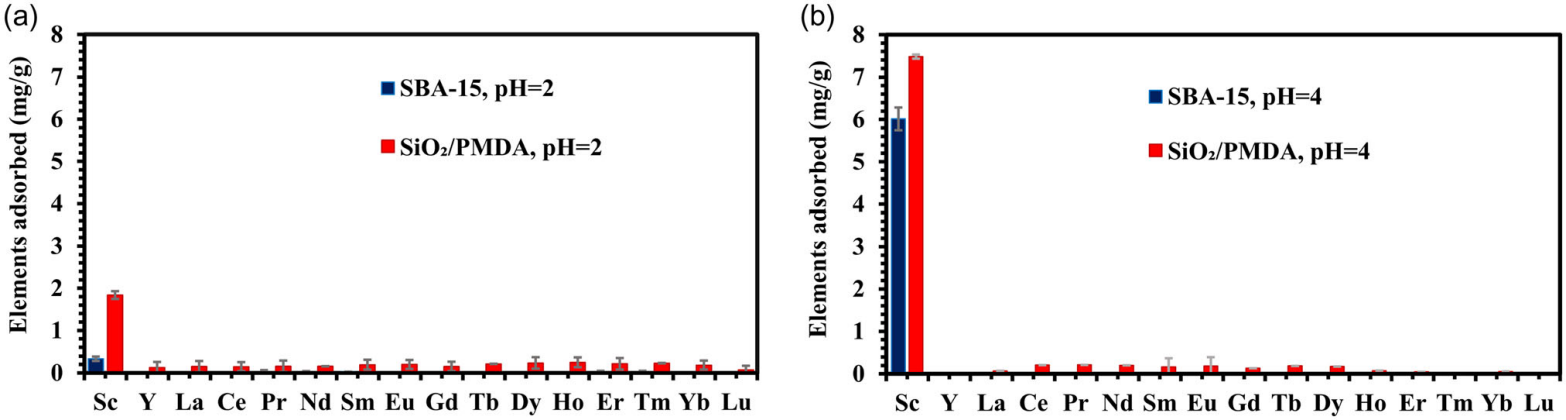
Adsorption of 16‐REE solutions by SiO_2_/PMDA and bare SBA‐15 at a) pH 2 and b) pH 4. Each element in the solution had a concentration of 6 mg L^−1^. Error bars represent the standard deviation of triplicate measurements.

**Figure** **6**a illustrates the selectivity profile when SiO_2_/PMDA‐modified silica and SBA‐15 were exposed to a pH 2 solution containing 16 REEs. It is evident that at higher pH (Figure 6b and Table S4), modified silica and SBA‐15 demonstrate significantly improved selectivity toward Sc ions, with adsorption capacities of ≈7.5 mg g^−1^ for modified silica and 6 mg g^−1^ for SBA‐15, compared to pH 2 (Figure 6a). The calculated separation factors indicate exceptional separation of Sc from other 15 REEs by both sorbents (Table S4). The reduced adsorption performance at pH 2, relative to pH 4 for ligand‐functionalized silica and SBA‐15, can be attributed to the material surfaces acquiring a positive charge as shown in Figure S6. This positive charge likely repels positively charged metal ions via electrostatic forces, aligning with findings from other studies.^[^ ^57^ ^]^ At the elevated pH, a ligand‐functionalized silica surface loses the positive charge and gains a strongly negative charge (Figure S6) that can attract the REE cations from the solution. The observed trend, wherein lower pH levels result in reduced adsorption while higher pH levels enhance adsorption, suggests that Sc ion adsorption onto ligand‐functionalized silica primarily occurs through coordination with deprotonated carboxyl groups, interacting with Sc^3+^ and ScOH^2+^ species available at these pH levels (Figure S10). Conversely, if carbonyls were also contributing to the adsorption of Sc ions through the chelation, the adsorption capacity of modified silica at pH 2 would likely be higher, as it will be shown for thorium ions below. Furthermore, the formation of chelates by carbonyl groups is likely more “suited” to larger ions like Nd, La, and Dy, given their larger ionic radii. This aligns with previous findings^[^ ^22^, ^51^ ^]^ that suggest chelation as one of the main mechanisms for these larger ions.

**Figure 7:**
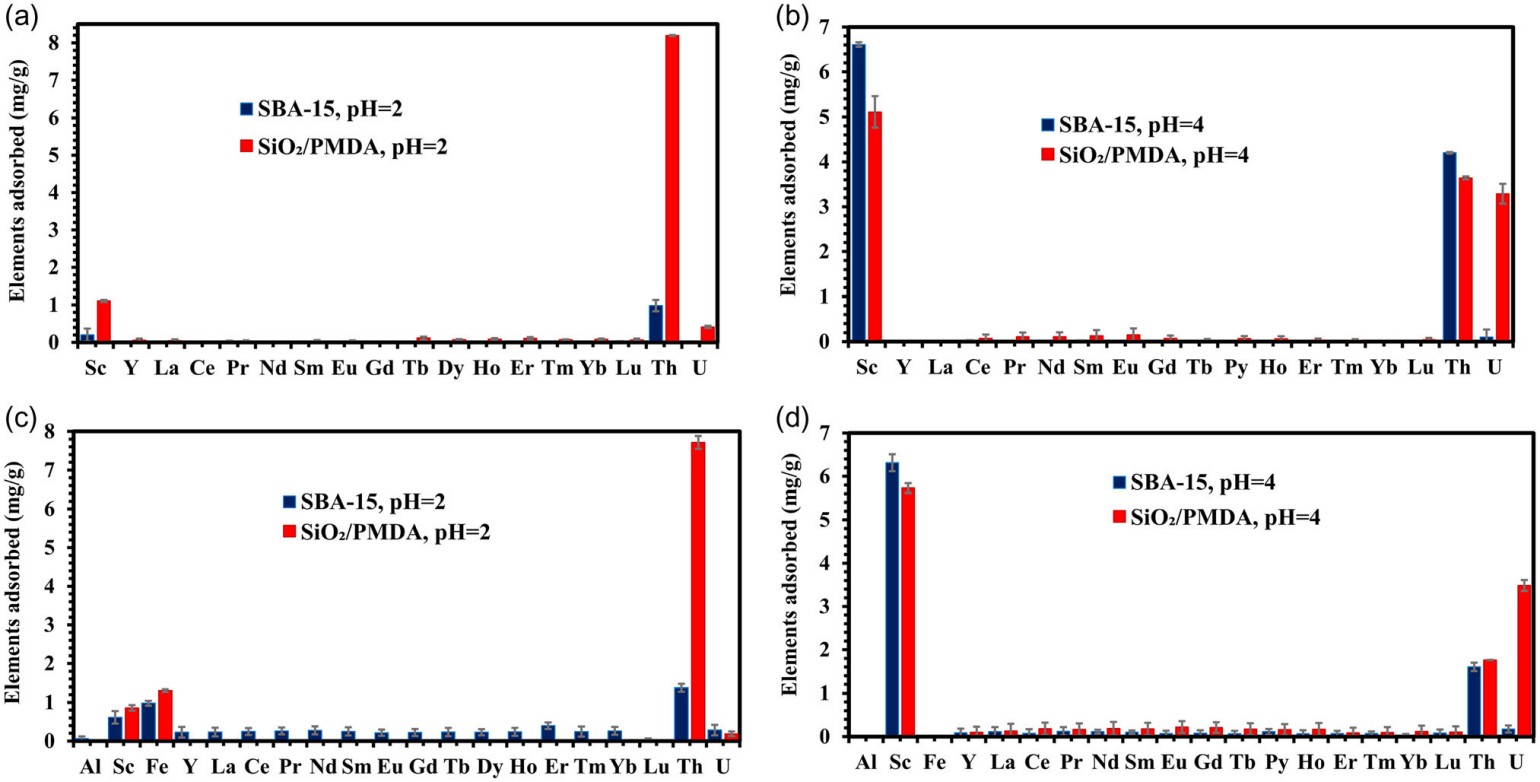
Adsorption of 18‐REE + U + Th and 20‐REE + U + Th + Fe + Al solutions by SiO_2_/PMDA and bare SBA‐15 at a,c) pH 2 and b,d) pH 4. Each element in the solution had a concentration of 6 mg L^−1^. Error bars represent the standard deviation of triplicate measurements.

**Figure** **7**a displays the selectivity profile for both sorbents in a pH 2 solution containing 16 REE elements, along with radioactive Th and U. It is observed that SiO_2_/PMDA‐modified silica exhibits excellent selectivity toward Th ions, achieving an adsorption capacity of ≈8.2 mg g^−1^, indicating that in solutions containing Th ions, SiO_2_/PMDA‐modified silica shows a strong preference for Th. The adsorption capacity for Sc and uranium ions for SiO_2_/PMDA‐modified silica is low at this pH level. The adsorption behavior of these elements by SiO_2_/PMDA‐modified silica changes at pH 4, as shown in Figure 7b. At this pH, Th adsorption decreases, while Sc adsorption increases, indicating a higher affinity for Sc. Additionally, the SiO_2_/PMDA‐modified silica demonstrates significant adsorption affinity toward U ions.

The decrease in Th ion adsorption at higher pH levels aligns with expectations, considering the significant reduction in the concentration of soluble Th ions beginning around pH 3.^[^ ^20^, ^58^ ^]^ The excellent selectivity toward Th ions by the SiO_2_/PMDA‐modified silica at pH 2 indicates that Th is primarily adsorbed through the chelation of Th ions by carbonyl groups. This conclusion stems from the observation that at pH 2, only a limited number of carboxyl groups on the modified silica surface are deprotonated according to the surface charge measurements (Figure S6). This reduces the possibility of ionic interactions between COO^–^ groups and Th ions while the adsorption capacity remains excellent at this pH. Therefore, at pH 4, the continued adsorption of Th ions by the SiO_2_/PMDA‐modified silica is likely due to the persistent affinity of the carbonyl groups for Th ions, an affinity that is unlikely to change at higher pH levels.

For uranium, it primarily exists in the form of UO_2_^2+^ ions at pH 2, while at pH 4, the solution also includes some amount of UO_2_(OH)^+^ ions (Figure S11). This variation implies that at pH 2, the carbonyl groups on the SiO_2_/PMDA‐modified silica surface contribute little to the adsorption of UO_2_^2+^ ions (as shown in Figure 7a). This conclusion is drawn from the fact that carbonyl groups are not affected by pH changes and remain intact at pH 2. If these groups were effectively chelating UO_2_^2+^ ions, a higher adsorption capacity at this pH would be expected. Conversely, at pH 4, the increased number of deprotonated carboxyl groups on the modified silica correlates with a significant increase in the adsorption capacity for U ions (Figure 7b), suggesting that these groups play a more active role in U adsorption at this pH. Thus, the increase in uranium ion adsorption capacity at pH 4 can be attributed to two primary factors: first, the presence of more deprotonated carboxyl groups, COO^–^, which provide additional adsorption sites for all forms of uranium ions present at this pH, i.e.,UO_2_^2+^ and UO_2_(OH)^+^, and second, the specific adsorption of the newly emerged UO_2_(OH)^+^ ions at pH 4 through chelation by carbonyl groups. Overall, deciphering the adsorption mechanism of uranium ions at different pH levels is complex, as two parameters change simultaneously in both solutions–the number of deprotonated COOH groups and the chemical nature of uranium species.

Contrasting with SiO_2_/PMDA‐modified silica, bare SBA‐15 at pH 2 displays poor adsorption behavior, as depicted in Figure 7a. The material shows enhanced adsorption for Sc and Th ions at pH 4 (Figure 7b), yet the practical effectiveness of thorium recovery at this pH is limited due to the reduced number of soluble Th ions available for adsorption.

The introduction of Al and Fe ions into the pH 2 solution with SiO_2_/PMDA‐modified silica did not significantly alter the adsorption behavior of the modified sorbent, as depicted in Figure 7a,c. Iron (Fe) ions were adsorbed to a similar extent as Sc ions, indicating a low adsorption capacity for both. Notably, the selectivity of SiO_2_/PMDA for Th ions remained excellent in this solution, as shown in Figure 7c and supported by the separation factors in Table S5, making it an excellent material for the selective extraction and isolation of thorium from complex mixtures. The adsorption capacity for Th remained almost unchanged compared to the pH 2 solution without Fe and Al ions (Figure 7a).

The addition of Al and Fe ions to the pH 4 solution did not affect the performance of the SiO_2_/PMDA, as shown in Figure 7b,d. It should be noted that at pH 4, Fe^3+^ species undergo hydrolysis (Figure S12), precipitating from the solution and making the adsorption of the target element more favorable. However, species such as Fe(OH)^2+^ and Fe(OH)^2+^ remain in solution and can hinder the adsorption of the target element. Despite this, SiO_2_/PMDA shows no affinity for these Fe species and preferentially adsorbs Sc and U (Figure 7d). The reduction in Th adsorption capacity at this pH was observed, likely due to a decrease in the concentration of soluble Th ions. The separation factors between U and the other 18 elements achieved by PMDA‐modified silica (Table S6) are very high, confirming the effectiveness of the developed material for the selective extraction and isolation of U from complex mixtures. This is consistent with its behavior in solutions absent of Al and Fe ions (Figure 7b).

Building on these findings, the comprehensive selectivity studies focusing on different multielement complex solutions (Figure 5, 6, 7) underscore the dual advantages of the SiO_2_/PMDA‐modified silica: its capability for selective extraction of Th, U, and Sc ions, and its ability to effectively separate these elements from REEs themselves. The latter capability is especially significant, as it addresses the critical challenge of separating and recovering REEs, a crucial aspect in advancing the field. In addition to the excellent selectivity exhibited by the PMDA‐modified silica, the sorbent maintained consistent adsorption capacity over ten reuse cycles (Figure S13a), even with incomplete desorption in each cycle (Figure S13b). The incomplete desorption suggests a strong interaction between Sc ions and the sorbent's functional groups, making it difficult to break the bonds between Sc and the sorbent.

The addition of Al and Fe ions to the pH 2 solution did not significantly impact the adsorption behavior of SBA‐15 for Th and Sc ions, as shown in Figure 7a,c. This result aligns with the sorbent's generally low performance in acidic environments. Specifically, at pH 2, it exhibited limited effectiveness in the 16‐element solution containing only REEs (Figure 6a), and this trend persisted in the 18‐element solution with REEs, U, and Th (Figure 7a), as well as in the 20‐element solution with the inclusion of Al and Fe (Figure 7c). Nevertheless, it is important to highlight the selective affinity of bare SBA‐15 for Sc ions over U ions at pH 4 (Figure 7b,d) which introduces the possibility of using SBA‐15 in a sequential separation strategy of these two elements. Initially, the extraction of Sc and U can be effectively performed by PMDA‐modified silica. Following this, SBA‐15 can be utilized to selectively remove Sc from the mixture, thereby enhancing the purity of the U fraction extracted by the PMDA‐modified silica. Although not directly tested in this work, this concept offers a promising avenue for our future research focused on the refined separation of these critical elements. Similar to SiO_2_/PMDA, SBA‐15 demonstrated a good reusability profile (Figure S13c), maintaining its adsorption capacity across 10 cycles of reuse. In contrast to modified silica, bare SBA‐15 showed full desorption on each cycle (Figure S13d), which may indicate weaker interactions between Sc ions and the functional groups of SBA‐15.

## 3 Conclusion

This study investigates the selective extraction of Sc, other REEs, and radionuclides like Th and U, using a novel ligand‐functionalized mesoporous silica. This innovative material proves to be an effective sorbent across a spectrum of multi‐element solutions which vary in acidity and complexity, containing anywhere from two to an array of elements. The functionalized mesoporous silica sorbent was developed via a simple two‐step modification process, utilizing mesoporous silica as the base matrix and pyromellitic dianhydride as the grafting ligand. A comprehensive solid‐state NMR analysis has not only confirmed the successful grafting of PMDA onto the mesoporous silica surface but has also elucidated the sorbent's structure shedding light on the possible mechanism of interaction of sorbent active sites with metal ions. Textural characteristics and morphological peculiarities of both sorbents were extensively investigated and determined using a suite of physicochemical techniques. The advanced functionalized mesoporous sorbent displayed remarkable selectivity for Sc ions at pH 4 across diverse solutions with 3, 4, 16, 18, and 20 elements, regardless of the number of REEs and other elements present in a solution. In Sc‐ion‐free REE solutions, it exhibited excellent adsorption capacity for other REEs, such as Nd, Dy, and La with a given preference for Nd. Additionally, this innovative material demonstrated a good affinity for U ions in solutions with 18 and 20 elements at pH 4. Upon lowering the pH to 2, the modified sorbent exhibited outstanding selectivity for Th ions. This wide‐ranging effectiveness, coupled with its good recyclability, highlights the potential of SiO_2_/PMDA‐modified silica for diverse industrial applications. Future work will focus on refining the separation process between U and Sc.

## Conflict of Interest

The authors declare no conflict of interest.

## Author Contributions

**Iryna Protsak**: Conceptualization (lead); Data curation (lead); Formal analysis (lead); Funding acquisition (lead); Investigation (lead); Methodology (lead); Project administration (lead); Resources (equal); Validation (lead); Visualization (lead); Writing—original draft (lead); Writing—review and editing (lead). **Martin Stockhausen**: Formal analysis (supporting); Resources (equal); Validation (supporting); Writing—review and editing (equal). **Aaron Brewer**: Data curation (supporting); Formal analysis (supporting); Funding acquisition (lead); Validation (supporting); Visualization (supporting); Writing—review and editing (equal). **Martin Owton**: Formal analysis (supporting); Methodology (supporting); Supervision (supporting); Validation (supporting); Writing—review and editing (equal). **Thilo Hofmann**: Funding acquisition (lead); Resources (lead); Validation (supporting); Formal analysis (Supporting); Writing—review and editing (equal). **Freddy Kleitz**: Conceptualization (supporting); Funding acquisition (equal); Project administration (supporting); Resources (lead); Validation (supporting); Formal Analysis (supporting); Writing—review and editing (equal).

## Supporting information

Supplementary Material

## Data Availability

The data that support the findings of this study are openly available in Open Science Framework at https://doi.org/10.17605/OSF.IO/UKPDS, reference number 0.
