# Supplementary material for: Advancing Selective Extraction: A Novel Approach for Scandium, Thorium, and Uranium Ion Capture
Source: Small Sci. 2024 Aug 28;4(10):2400171. doi: 10.1002/smsc.202400171 (PMC11935155; doi:10.1002/smsc.202400171)
Supplement: Supplementary file 1 — Supplementary Material [file SMSC-4-2400171-s001.pdf]

Supporting Information

Advancing Selective Extraction: A Novel Approach for Scandium, Thorium, and Uranium Ion Capture

Iryna Protsak, Martin Stockhausen, Aaron Brewer, Martin Owton, Thilo Hofmann, and Freddy Kleitz**

1. Characterization

Low-angle powder diffraction data were recorded on a PANalytical Empyrean diffractometer (Malvern PANalytical, United Kingdom) in transmission geometry (Focusing mirror) using Cu $K\alpha_{1+2}$ radiation operated at a voltage of 45 kV, a tube current of 40 mA and with a fixed divergence slit of 0.76 mm. Measurements were performed in the continuous mode with a step size of 2θ of 0.013° and a data collection time per step of 50 s for the transmission mode.

Transmission electron microscopy (TEM) images were obtained using a FEI /TFS Tecnai G2 F20 FEGTEM at an accelerating voltage of 200 kV. The samples for TEM imaging were prepared by dropping a small amount of ethanol containing a suspended powder sample on a holey carbon film-coated 300 mesh copper grid.

Solid-state NMR spectra were measured on a Bruker Avance NEO 500 wide bore system (Bruker BioSpin, Ettlingen, Germany) using a 4 mm triple resonance magic angle spinning (MAS) probe with a resonance frequency of 125.78 MHz for ^{13}C and 99.38 MHz for 29Si, respectively. For ^{13}C the MAS rotor spinning speed was set to 14 kHz and the cross polarization (CP) contact time to 3 ms, for ^{29}Si to 8 kHz and 5 ms. A ramped contact pulse was used for CP and during acquisition ^{1}H was high-power decoupled using SPINAL with 64 phase permutations. The chemical shifts are reported in ppm and are referenced external for ^{13}C to adamantane by setting the low field signal to 38.48 ppm, and for ^{29}Si to DSS by setting the signal to 0 ppm.

Organic elemental analysis was performed using an EA3000 CHNS-O elemental analyzer (Eurovector). The suitability of the method for the characterization of modified silica material has been studied and verified before [1]. Each value is the average of three replicate measurements which showed excellent reproducibility.

Fourier-Transform Infrared (FTIR) spectra were recorded using a Cary 360 (Agilent) with a single reflection ATR (attenuated total reflectance)-cell. Each spectrum was recorded by averaging 32 scans and applying a *two*-fold zero filling. The resulting spectral resolution is about 1 cm^{-1}. The presented figures show an average from three different measurements on different equivalents of the sample.

Thermogravimetric analysis (TGA) was performed on a Netzsch instrument (NETZSCH STA 449F3) from 25 to 800 °C at a heating rate of 10 °C min^{-1} under an O_2/N_2 atmosphere. The mass losses (%) were estimated in the temperature range from 150 to 800 °C.

N_2-physisorption isotherm measurements were measured at −196 °C using an Autosorb-$_i$$Q_2$ sorption analyzer (Anton Paar, Boynton Beach, FL, USA). All samples were degassed under vacuum at 150 °C for 14 h before measurement. The specific surface area was calculated calculated using the Brunauer-Emmet-Teller (BET) method in the relative pressure range 0.05–0.3 P/P_o. The pore size distribution plot was obtained from the equilibrium branch of the

isotherms by the NLDFT method (silica with cylindrical pore geometry). The calculations were performed using the ASiQwin 5.21 software provided by Anton Paar Quantatech Inc.

The *zeta-potential measurements* of SBA-15 and SiO_2/PMDA were conducted using a Malvern Nano Zetasizer ZS. To ensure accurate calibration before the measurements, a standard suspension containing carboxylate-modified polystyrene latex microspheres with a zeta potential of -40 (±6) mV was utilized. Aqueous suspensions of the materials (0.7 mg/mL) were prepared through ultrasonic bath treatment lasting 45 minutes. The zeta-potential values were then determined by analyzing the supernatants.
Inductively Coupled Plasma Optical Emission spectroscopy (ICP-OES) and Inductively Coupled Plasma Mass Spectrometry (ICP-MS) measurements were conducted using an Agilent 5110 ICP-OES and Agilent 7900 ICP-MS respectively.

The conditions for the ICP-OES measurements were defined as follows: Read time: 5 s, RF power: 1.2 kW, stabilization time: 8 s, viewing mode: Axial, viewing height: 8 mm, nebulizer gas flow: 0.65 l/min, plasma flow: 12 l/min, Aux flow: 1.2 l/min.

ICP-MS measurements were performed in no-gas mode. The operation parameters for the plasma were set to the following values: RF power: 1550 W, RF matching: 1.80 V, sample depth: 10 mm, nebulizer gas flow: 0.8 l/min, dilution gas: 0.4 l/min. Parameters for data acquisition were as follows: acquisition mode: spectrum, sweeps/replicate: 80, replicates: 3, integration time/ mass: 0.1 sec.

2. Materials and methods

2.1 Materials

Tetraethyl orthosilicate (TEOS, 98%), anhydrous toluene (99.8%), anhydrous tetrahydrofuran (99.5%, THF), anhydrous methanol (99.8 %, CH_3OH), poly(ethylene glycol)-*block*-poly(propylene glycol)-*block*-poly(-ethylene glycol) (Pluronic P123, $EO_{20}PO_{70}EO_{20}$, $M_n \sim 5800$), sodium hydroxide (NaOH, 98%) flakes were purchased from Sigma Aldrich. Technical grade ethanol (C_2H_5OH, 96%) was purchased from Brenntag Austria GmbH. 3-Aminopropyltriethoxysilane (APTES, 98%), and triethylamine (TEA, 99%) were purchased from Alfa Aesar. Hydrochloric acid (HCl, 37%) was purchased from VWR Chemicals. Pyromellitic dianhydride (PMDA, 97%) was purchased from Fluorochem Ltd. Super pure nitic acid (HNO_3, 67-70%) and rare earth elements (REEs) stock (18 elements,1800 ppm) containing uranium and thorium were purchased from Carl Roth. Rare earth elements stock (16 elements, 800 ppm), aqueous scandium (10,000 ppm), neodymium (10,000 ppm), dysprosium (1000 ppm), and lanthanum (1000 ppm) were purchased from LabKings. Aqueous aluminum (1000 ppm) and iron (1000 ppm) were purchased from Inorganic Ventures.

2.2 Synthesis of mesoporous SBA-15 silica

SBA-15 mesoporous silica was synthesized using the procedure outlined by Guillet-Nicolas et al. [2]. In brief, 8.072 grams of Pluronic P123 were first dissolved in a solution consisting of concentrated HCl (8.025 grams, 37%) and distilled water (146.25 grams). This mixture was stirred at 30°C at a rate of 500 rpm overnight until the polymer was completely dissolved. Once the P123 was fully dissolved, 17.428 grams of TEOS were introduced into the mixture, and it was left under stirring at 30 °C overnight. After 24 hours, the solution obtained underwent hydrothermal treatment in an autoclave, utilizing Teflon containers for 48 hours at 140 °C. Subsequently, the resulting product was subjected to vacuum filtration using Whatman® filter paper (diameter 125 mm) and then dried at room temperature. The resulting white powder was transferred into crucibles and dried once more at 140 °C for 12 hours in a muffle furnace. After thorough drying, the powder was placed in 250 ml polypropylene (PP) bottles. Ethanol was added until one-third of the bottle was filled, followed by the addition of 1-3 drops of HCl (37%). The resulting suspension was stirred for an additional 45 minutes at a speed of 500 rpm. The solution was then vacuum-filtered and dried. Finally, the template was removed through the calcination of the obtained powder at 550 °C for 5 hours.

2.3 Synthesis of the novel mesoporous sorbent - SiO_2/PMDA

2.3.1 Synthesis of amino-functionalized SBA-15

SBA-15 silica was functionalized with APTES to introduce -NH_2 functionality to the pore surface. The functionalization with APTES followed a modified protocol reported in Iriarte-Mesa et al. [3] Typically, 0.5 grams of SBA-15 were degassed overnight at 150 °C and then dispersed in 15 mL of anhydrous toluene under stirring in an argon atmosphere at 115 °C. Subsequently, 1 mL of APTES was added, and the reaction mixture was stirred overnight, for at least 22 hours, at 115 °C. The amino-functionalized SBA-15, denoted as SiO_2_NH_2, was collected by centrifugation (7500 rpm × 15 minutes), subjected to two washing steps with toluene and two washings with ethanol, and then dried at 85 °C overnight.

2.3.2 Functionalization of SiO_2_NH_2 with PMDA

The amino-functionalized SBA-15 was further functionalized with pyromellitic dianhydride using a new procedure described in Scheme S1. Typically, 0.5 grams of pre-dried SiO_2_NH_2, which had been dried at 60 °C overnight, were dispersed in 10 ml of anhydrous THF under an argon atmosphere at room temperature. Separately, 0.5 grams of PMDA were dissolved in 15 ml of anhydrous THF under an argon atmosphere. Next, 642 µl of TEA was added to the SBA-15_NH_2 solution, followed by the addition of the dissolved PMDA solution. The reaction mixture was heated to 50°C and allowed to stir for at least 22 hours under an inert atmosphere. The resulting powder was collected by centrifugation (7500 rpm × 15 minutes) and underwent 3-4 washing steps with a mixture composed of 80 ml of THF, 20 ml of

distilled water, 284 µl of HCl, and 1 washing with technical-grade ethanol. The addition of acid served to catalyze the formation of carboxyl groups on the silica surface and within the pores. Subsequently, the powder was dried at 80 °C overnight. Additionally, the freshly synthesized powders were washed with methanol (100 ml)/HCl (284 µl) and ethanol (100 ml)/HCl (284 µl) to investigate the structure of the grafted anhydride on the silica surface.

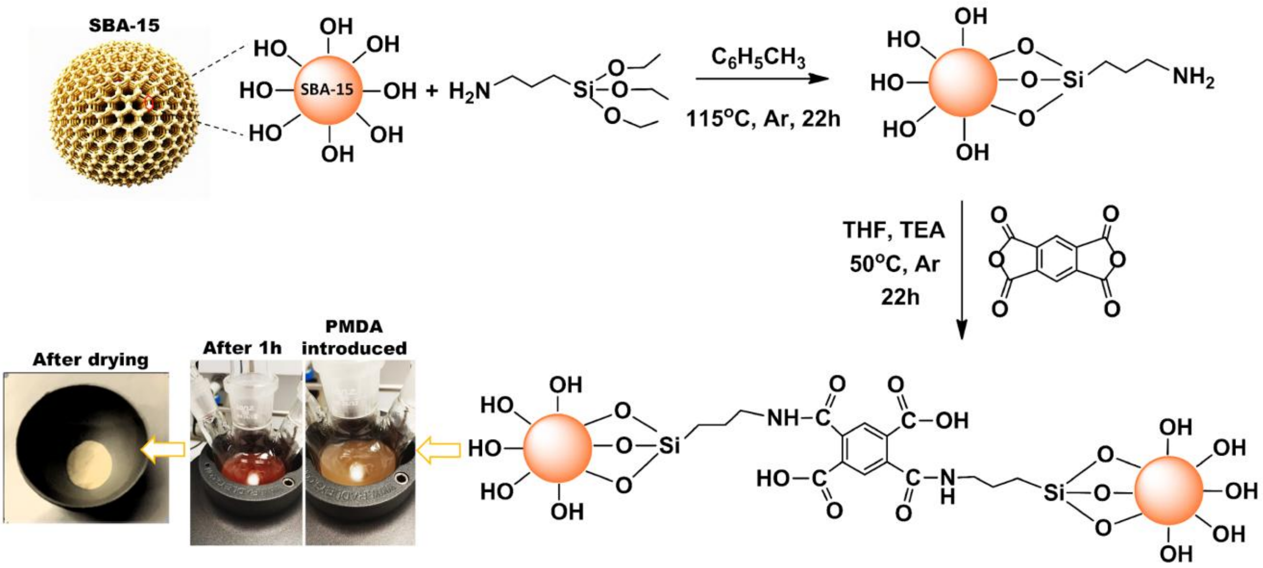

Scheme S1. Schematic representation of the two-step modification process applied to the surface of SBA-15 silica to generate the mesoporous sorbent. Also included are photographs depicting the material and reaction media.

2.4 Extraction studies

To construct adsorption isotherms, 10.1 mg aliquots of the native SBA-15 silica and SiO_2/PMDA powder were exposed to 14.8 ml of Sc or Nd solution. Solutions were adjusted to pH=4 and kept at room temperature.
The extraction capacity at equilibrium was calculated by the given equation (S1):

$$q_e = \frac{C_0 - C_e}{m} \times V \qquad \text{(S1)}$$

C_o – represents the initial measured concentration of ions (mg/L); C_e represents equilibrium concentration of ions (mg/L); m represents mass of the sorbent (mg); V is the volume of the solution (L).
The obtained isotherms for Sc and Nd were fitted with typical Langmuir (equation S2) and Freundlich (equation S3) isotherm models using *non*-linear regression:

$$q_e = q_{max} * \frac{K_L * C_e}{1 + K_L * C_e} \qquad \text{(S2)}$$

where C_e represents the equilibrium concentration of ions (mg/L); q_m is the maximum adsorption

capacity (mg/g); K_L is the Langmuir binding constant (L/g); and q_e is the equilibrium adsorption capacity (mg/g):

$$q_e = K_L * C_e \wedge (\frac{1}{n}) \qquad \text{(S3)}$$

$K_{F.}$ is the Freundlich empirical constant ((mg/g)/(L/mg)^$^{(1/n)}$) and n is the dimensionless nonlinearity parameter associated with the intensity of the adsorption.

For the kinetics tests, 10.1 mg aliquots of the sorbents were exposed to 14.8 mL of a solution containing Sc (54 mg/L) at pH=4. The samples were agitated at room temperature for specific durations, ranging from 0 to 1250 minutes before the experiment was concluded.
The sorption kinetics of the newly synthesized materials were calculated using the pseudo-second-order (S4) kinetic model, which provided a good fit to the experimental data:

$$\frac{d(q_t)}{d(t)} = \mathrm{k} \times (q_e - q_t)^{\wedge 2} \qquad \text{(S4)}$$

where q_t is the amount of solute adsorbed at time t (mg/g), q_e is the equilibrium adsorption capacity (mg/g), k is the rate constant of pseudo-second-order adsorption (g/(mg min)).

For the selectivity tests involving REEs (16 elements), REEs+U+Th (18 elements), and REEs+U+Th+Fe+Al (20 elements), 10.1 mg aliquots of both sorbents were exposed to 14.8 mL of mixed solutions containing 6 ppm of each element. The tests were conducted at pH=2 and pH=4.
For the selectivity tests involving Sc/La/Nd/Dy, Sc/Nd/Dy, La/Nd/Dy, and Nd/Dy (combinations of 4, 3, and 2 elements), 10.1 mg aliquots of both sorbents were exposed to 14.8 mL of mixed solutions containing 34 ppm of each element. These tests were conducted at pH=4.
All samples were agitated at room temperature for 10,080 minutes before the experiment was concluded. In all cases, liquid-phase samples were diluted in 3% nitric acid, and their contents in Sc, Nd, and other Rare Earth Elements (REEs), as well as U, Th, Al, and Fe, were analyzed using either an Agilent 5110 ICP-OES or an Agilent 7900 ICP-MS. The choice between these two methods depended on the concentration range being studied and the potential interference of elements in the mixed multi-element solutions. Before ICP-OES analysis, the solutions were centrifuged for 30 minutes at 8000 rpm and then the dilution was performed, while prior to ICP-MS analysis, the samples were centrifuged and filtered. All experiments were conducted in triplicate.

For the re-use tests, 10.1 mg of SiO_2/PMDA or SBA-15 powder suspended in ethanol/water solution was packed inside a small Econo-Column® (0.5 × 5 cm, Bio-Rad Laboratories Ges.m.b.H., Austria). Before loading the extraction solution, the powder was conditioned with 5 ml of diluted nitric acid (pH=4). The Sc extraction solution (162 ppm for SiO_2/PMDA and 140 ppm for SBA-15, 5 mL) was passed through the column using a peristaltic pump (VWR® Peristaltic Pump) with a fixed flow rate of 1.2 mL/min. The retained element was eluted from

the column with 20 mL of 0.1 M solution of HNO_3. Afterward, the column was washed and reconditioned with diluted HNO_3 (5 mL, pH = 4) and used for the second extraction. The above-mentioned procedure was repeated 10 times.
The desorption was calculated as shown below:

$$D\ (\%) = \frac{d}{a} * 100\% \qquad \text{(S5)}$$

Where *d* represents the desorption amount (in mg) and *a* signifies the adsorbed amount of metal (in mg). In cycles where complete desorption was not achieved, the calculation of desorption efficiency took into account the residual carryover of metal ions from the previous cycle:

$$D\ (\%) = {d_n}\Big/{\left(a_n + \left(a_{(n-1)} - d_{(n-1)}\right)\right)} * 100\% \qquad \text{(S6)}$$

In this formula, d_n represents the desorption amount in the current cycle (mg), a_n is the adsorbed amount of metal in the current cycle (mg), and $a_{(n-1)}$ and $d_{(n-1)}$ account for the carryover of metal ions from the previous cycle, where *n* indicates the cycle number.

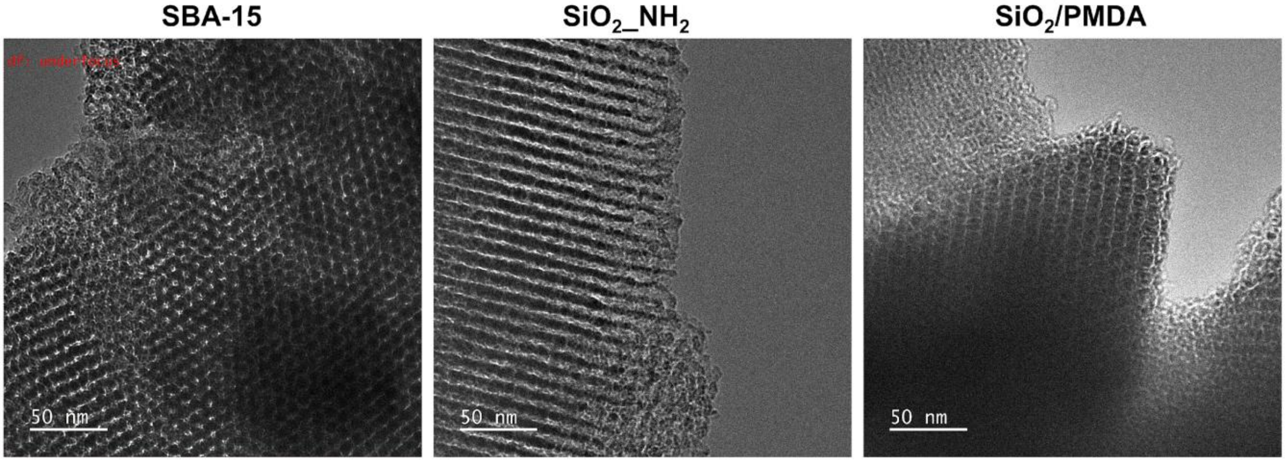

Figure S1. Representative TEM images of the studied samples, as indicated, recorded in under-focus mode.

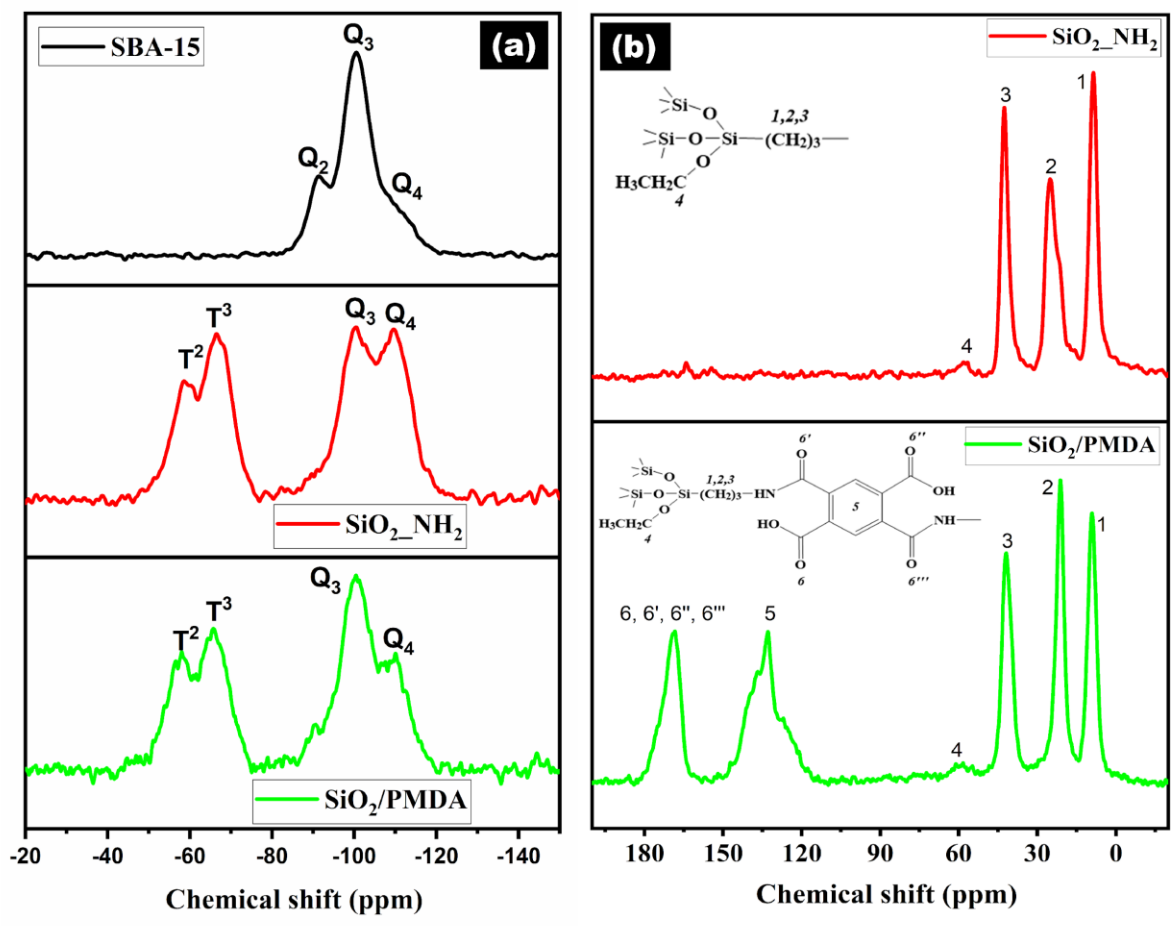

Figure S2. Solid-state ^{29}Si CP/MAS NMR spectra (*a*) and solid-state ^{13}C CP/NMR spectra (*b*) of the samples studied, as indicated.

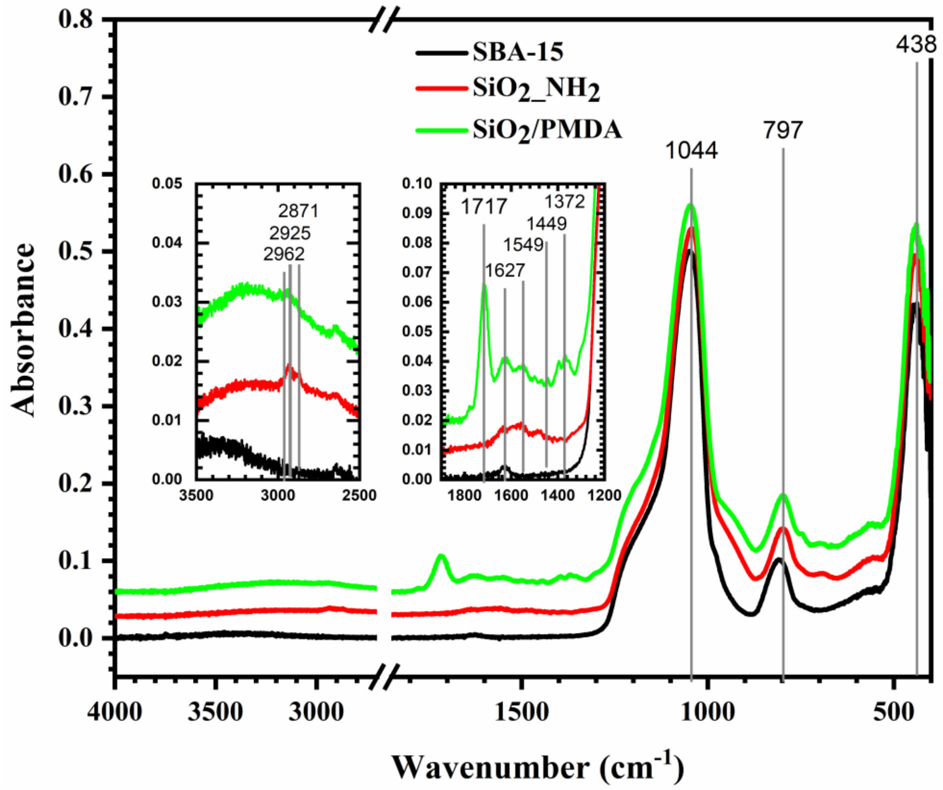

Figure S3. FTIR spectra of the studied samples as indicated. (*b*) Mass loss profile for the modified samples, as indicated.

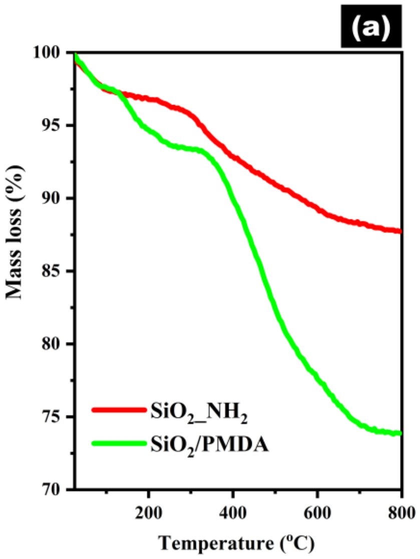

(b)

Materials	Mass loss[a], %	CHN (%)		
		%, C	%, H	%, N
SBA-15_NH_2	9.3	5.9	1.7	2.1
SiO_2/PMDA	22.5	12.9	1.8	1.8

Figure S4. (*a*) Mass loss profile for the modified samples, as indicated. (*b*) The table presents the mass loss observed via thermogravimetric analysis (TGA) in the temperature range of 150-800 °C, along with the carbon and nitrogen content quantified by elemental (CHN) analysis.

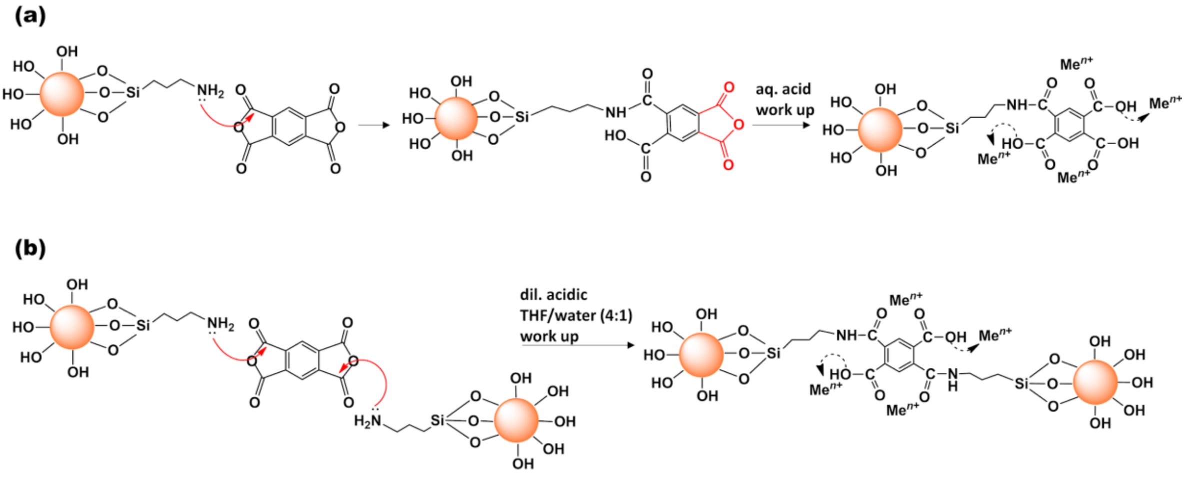

Scheme S2. Scheme depicting the two potential reaction *pathways* of SiO_2_NH_2 (amine-functionalized silica) with pyromellitic dianhydride.
Me^{n+}corresponds to metal ion.

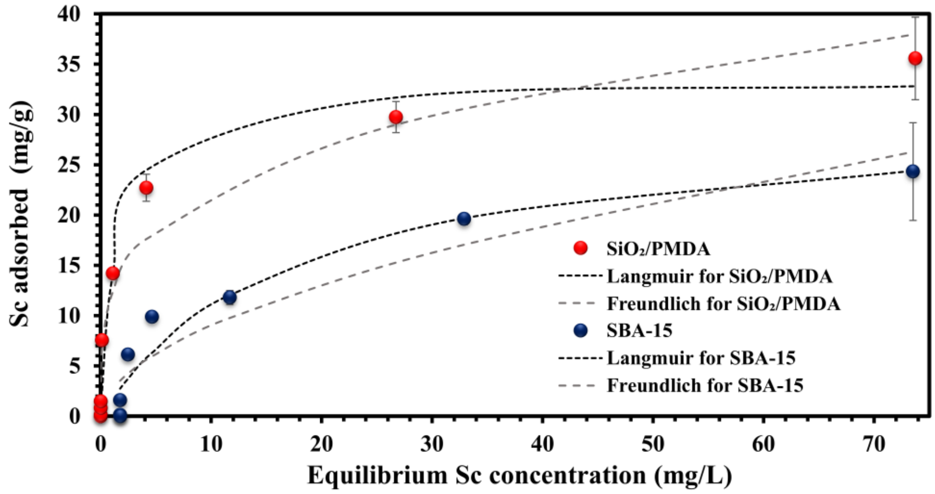

Figure S5. Scandium adsorption isotherms for SBA-15 and SiO_2/PMDA (measured in the concentration range of 0-100 mg/L), analyzed using the Langmuir and Freundlich models. All adsorption experiments were conducted at pH = 4 and a temperature of 25 °C. The error bars indicate the standard deviation from triplicate measurements.

Table S1. Equilibrium constants for both the Langmuir and Freundlich isotherm models, derived from the experimental data on the adsorption of Sc ions onto SBA-15 and SiO_2/PMDA surfaces.

Sorbents/Ions	Experimental	Langmuir model			Freundlich model		
SiO_2/PMDA	Max Q_e (mg/g)	Q_{max} (mg/g)	K_L (L/g)	R^2	K_F (mg/g)/(L/mg)^(1/n)	n	R^2
Sc^{3+}	35.6±4.1	33.5	0.7	0.97	12.1	3.8	0.95
SBA-15	Max Q_e (mg/g)	Q_{max} (mg/g)	K_L (L/g)	R^2	K_F (mg/g)/(L/mg)^(1/n)	n	R^2
Sc^{3+}	24.3±4.9	30.3	0.06	0.97	2.6	1.9	0.94

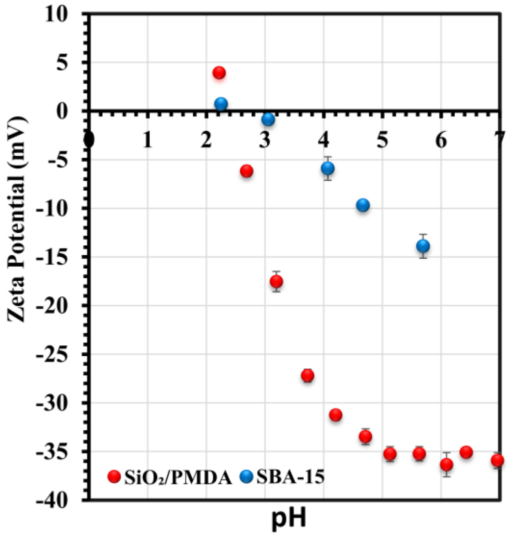

Figure S6. Zeta potential of synthesized samples, as indicated.

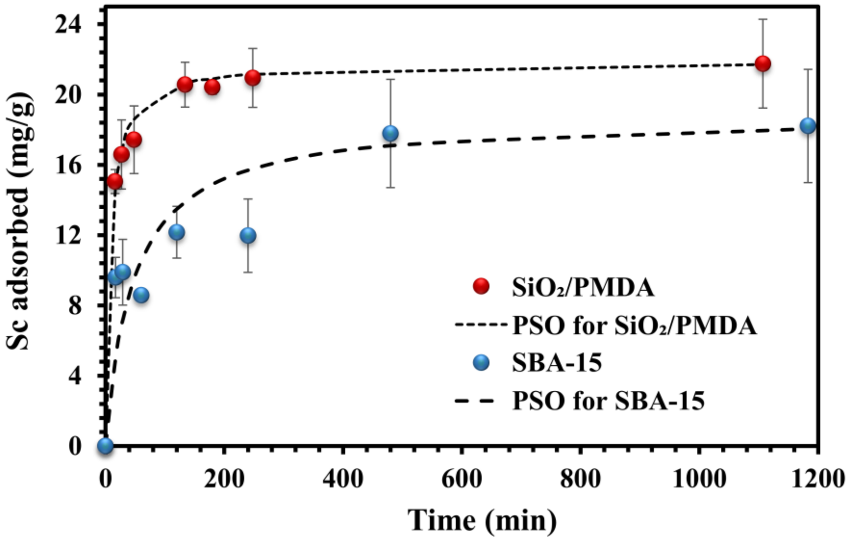

Figure S7. (*a*) The effect of contact time on Sc adsorption by SiO_2/PMDA and SBA-15 at pH 4 and at a temperature of 25 °C, with an initial Sc concentration of 54 mg/L for both samples. Data points represent the average of triplicate measurements, and the dash line indicates the fit to the pseudo-second-order (PSO) model.

Table S2. Estimated adsorption kinetics parameters for Sc adsorption onto both sorbents, derived from the experimental data and based on the pseudo-second-order fitting.

Sorbents/Ions	Experimental	PSO, Linear fit		
SiO_2/PMDA for *Sc^{3+}*	Qe(mg/g)	Model Qe (mg/g)	k_2 (g/(mg min))	R^2
	21.8±2.5	21.9	0.005	0.99
SBA-15 for *Sc^{3+}*	18.2±3.2	18.8	0.001	0.99

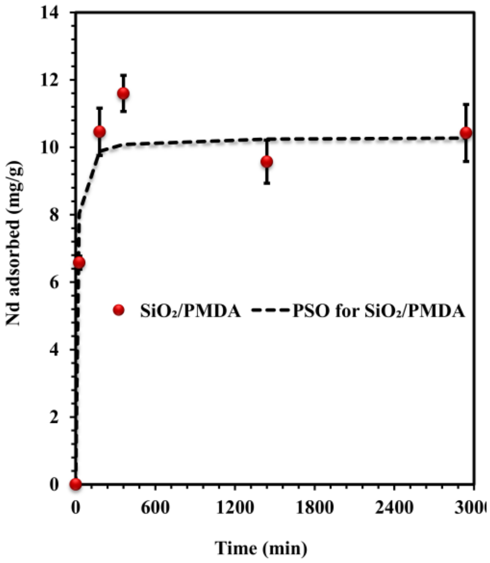

Figure S8. (*a*) The effect of contact time on Nd adsorption by SiO_2/PMDA at pH 4 and at a temperature of 25 °C, with an initial Nd concentration of 34 mg/L. Data points represent the average of triplicate measurements, and the dash line indicates the fit to the pseudo-second-order (PSO) model.

Table S3. Estimated adsorption kinetics parameters for Nd adsorption onto modified sorbent, derived from the experimental data and based on the pseudo-second-order fitting.

Sorbents/Ions	Experimental	PSO, Linear fit		
SiO_2/PMDA for Nd^{3+}	Qe(mg/g)	Model Qe (mg/g)	k_2 (g/(mg/min))	R^2
	10.4±0.85	10.3	0.01	0.99

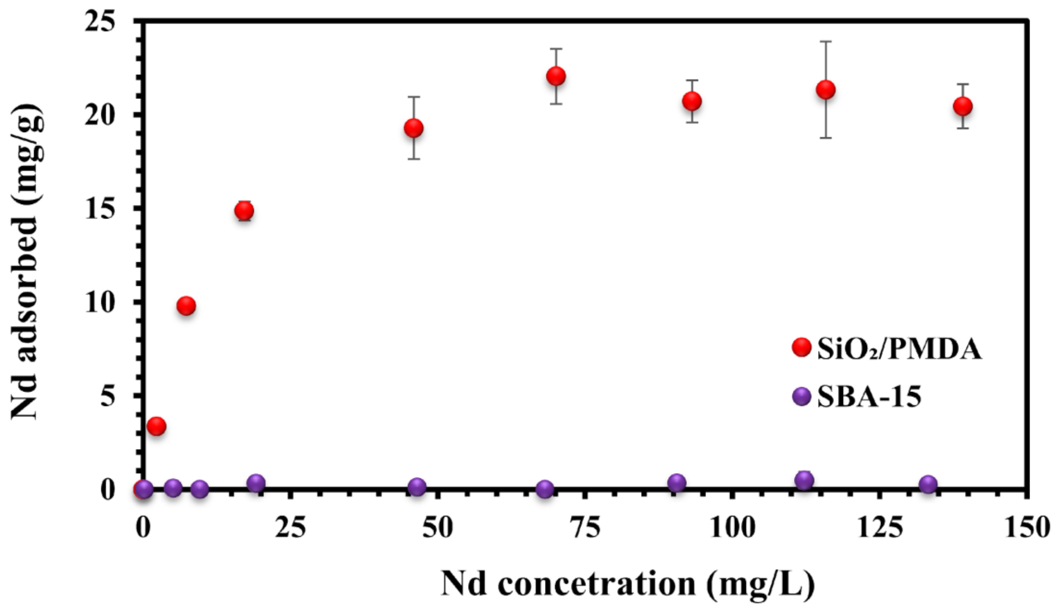

Figure S9. Neodymium adsorption profile for SBA-15 and SiO_2/PMDA powders. All adsorption experiments were conducted at a pH of 4 and a temperature of 25 °C in the concertation range of 0-150 mg/L. The error bars indicate the standard deviation from triplicate measurements.

Table S4. Adsorption capacities of adsorbed elements for PMDA-modified silica and non-modified silica, SBA-15, and separation factors for respective sorbents (the data derived from Figure 6*b*).

The symbol ∞ denotes that the adsorption of Sc is infinitely higher compared to the other element, as the other element showed 0 adsorption while Sc showed an adsorption capacity of 7.48±0.05 mg/g for SiO_2/PMDA and 6.02±0.27 for SBA-15.

Element	Adsorption capacity (a), mg/g, SiO_2/PMDA	stdev	Separation Factors for SiO_2/PMDA (α=a(Sc)/a(E))	Adsorption capacity (a), mg/g, SBA-15	stdev	Separation factors for SBA-15 (α=a(Sc)/a(E))
**Sc**	**7.48**	0.04786	-	**6.02**	0.26856	-
Y	0	0	7.48/0=∞	0	0	6.02//0=∞
La	0.07	0	106.86	0	0	6.02//0=∞
Ce	0.21	0	35.62	0	0	6.02//0=∞
Pr	0.21	0	35.62	0	0	6.02//0=∞
Nd	0.2	0	37.4	0	0	6.02//0=∞
Sm	0.17	0.20145	44	0	0	6.02//0=∞
Eu	0.18	0.20699	41.56	0	0	6.02//0=∞
Gd	0.13	0	57.54	0	0	6.02//0=∞
Tb	0.19	0	39.37	0	0	6.02//0=∞
Dy	0.18	0	41.56	0	0	6.02//0=∞
Ho	0.08	0	93.5	0	0	6.02//0=∞
Er	0.05	0	149.6	0	0	6.02//0=∞
Tm	0	0	7.48/0=∞	0	0	6.02//0=∞
Yb	0.06	0	124.67	0	0	6.02//0=∞
Lu	0	0	7.48/0=∞	0	0	6.02//0=∞

Table S4 shows excellent separation between Sc from the other 15 REEs at pH=4 by SiO_2/PMDA and SBA-15.

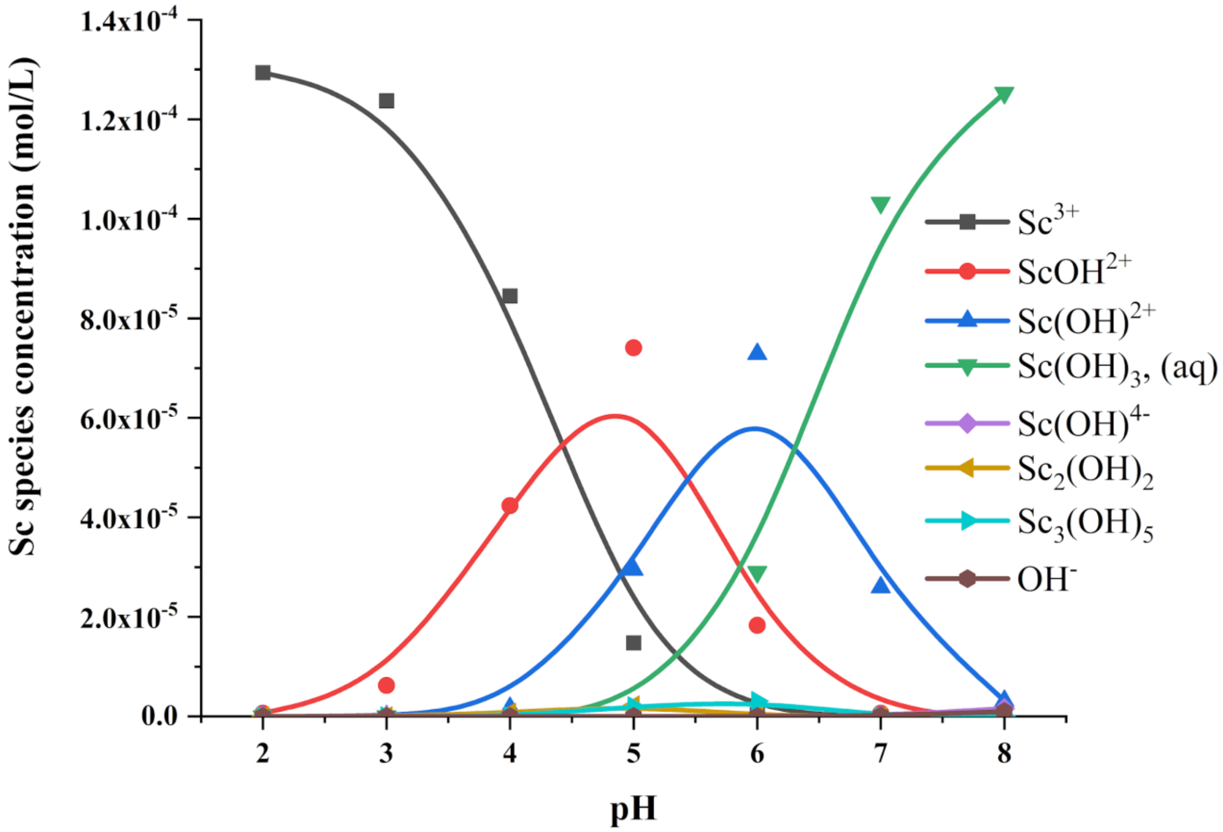

Figure S10. Distribution of Sc (III) species across different pH levels obtained with program ChemEQL V3.2.

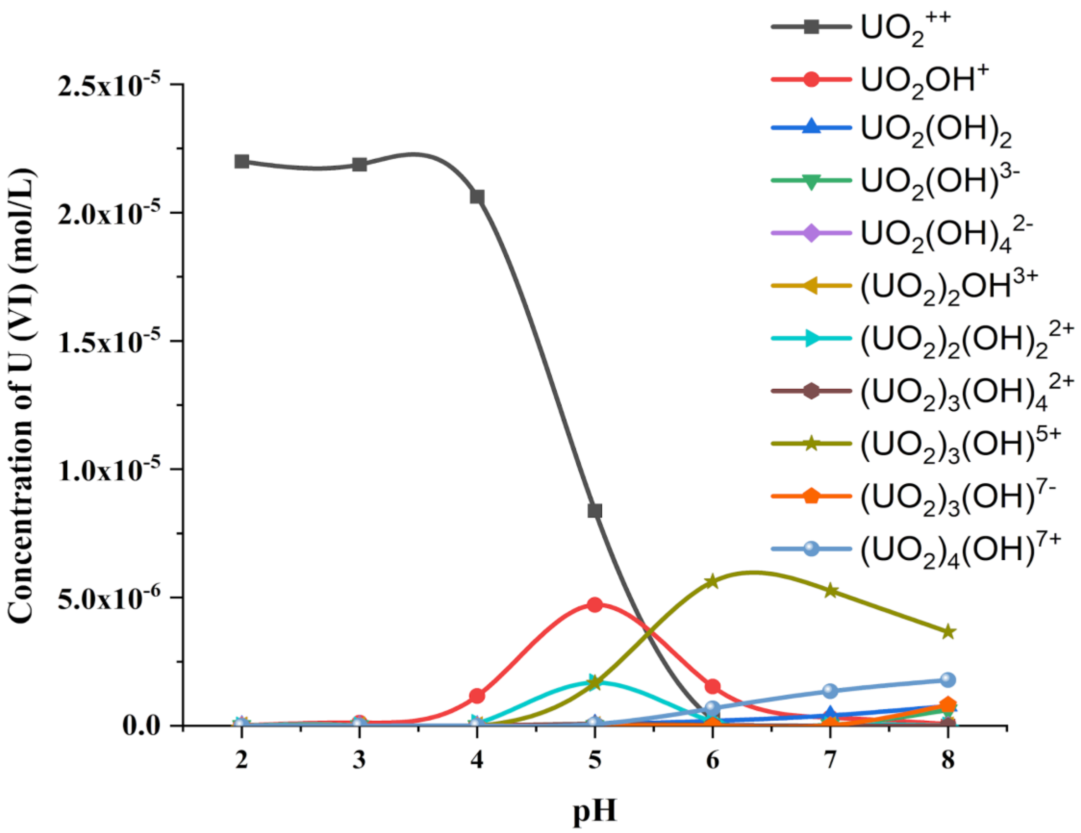

Figure S11. Distribution of U(VI) species across different pH levels obtained with program ChemEQL V3.2.

Table S5. Adsorption Capacities of adsorbed elements for modified silica and separation factors (derived from Figure 7*c*).

The symbol ∞ denotes that the adsorption of **Th** is infinitely higher compared to the other element, as the other element showed 0 adsorption while **Th** showed an adsorption capacity of 7.72±0.17 mg/g.

Element	Adsorption Capacity (a), mg/g, of SiO_2/PMDA at pH=2	Stdev	Separation Factors (α=a(Th)/a(E))
Al	0	0	7.72/0=∞
Sc	0.86	0.073387	8.9
Fe	1.31	0.035364	5.9
Y	0	0	7.72/0=∞
La	0	0	7.72/0=∞
Ce	0	0	7.72/0=∞
Pr	0	0	7.72/0=∞
Nd	0	0	7.72/0=∞
Sm	0	0	7.72/0=∞
Eu	0	0	7.72/0=∞
Gd	0	0	7.72/0=∞
Tb	0	0	7.72/0=∞
Dy	0	0	7.72/0=∞
Ho	0	0	7.72/0=∞
Er	0	0	7.72/0=∞
Tm	0	0	7.72/0=∞
Yb	0	0	7.72/0=∞
Lu	0	0	7.72/0=∞
**Th**	**7.72**	0.166165	-
U	0.19	0.062806	40.6

Table S5 shows the excellent separation of Th from the other 19 elements by SiO_2/PMDA, as indicated by the separation factor values.

Table S6. Adsorption Capacities of adsorbed elements for modified silica and separation factors (derived from Fig. 7*d*).

The symbol ∞ denotes that in some cases the adsorption of **U** is infinitely higher compared to the other element, as the other element showed 0 adsorption while **U** showed an adsorption capacity of 3.5±0.13 mg/g.

Element	Adsorption Capacity (a), mg/g, of SiO_2/PMDA at pH=4	Stdev	Separation Factors (α=a(U)/a(E))
Al	0	0	3.5/0=∞
Sc	5.73	0.111597	(Sc)5.73/ (U)3.5=1.64
Fe	0	0	3.5/0=∞
Y	0.1	0.127476	35
La	0.13	0.168568	26.92
Ce	0.18	0.141179	19.44
Pr	0.17	0.138192	20.59
Nd	0.19	0.151472	18.42
Sm	0.18	0.142019	19.44
Eu	0.22	0.136171	15.91
Gd	0.21	0.122341	16.67
Tb	0.17	0.136114	20.59
Py	0.16	0.127485	21.88
Ho	0.17	0.146973	20.59
Er	0.09	0.118011	38.89
Tm	0.09	0.121965	35
Yb	0.12	0.136732	29.17
Lu	0.1	0.132667	35
Th	1.76	0.003437	1.99
**U**	**3.5**	0.126487	-

Table S6 shows the excellent separation of U from the other 18 elements by SiO_2/PMDA, as indicated by the separation factor values. The only element that shows better adsorption than U is Sc, as indicated in Table S6.

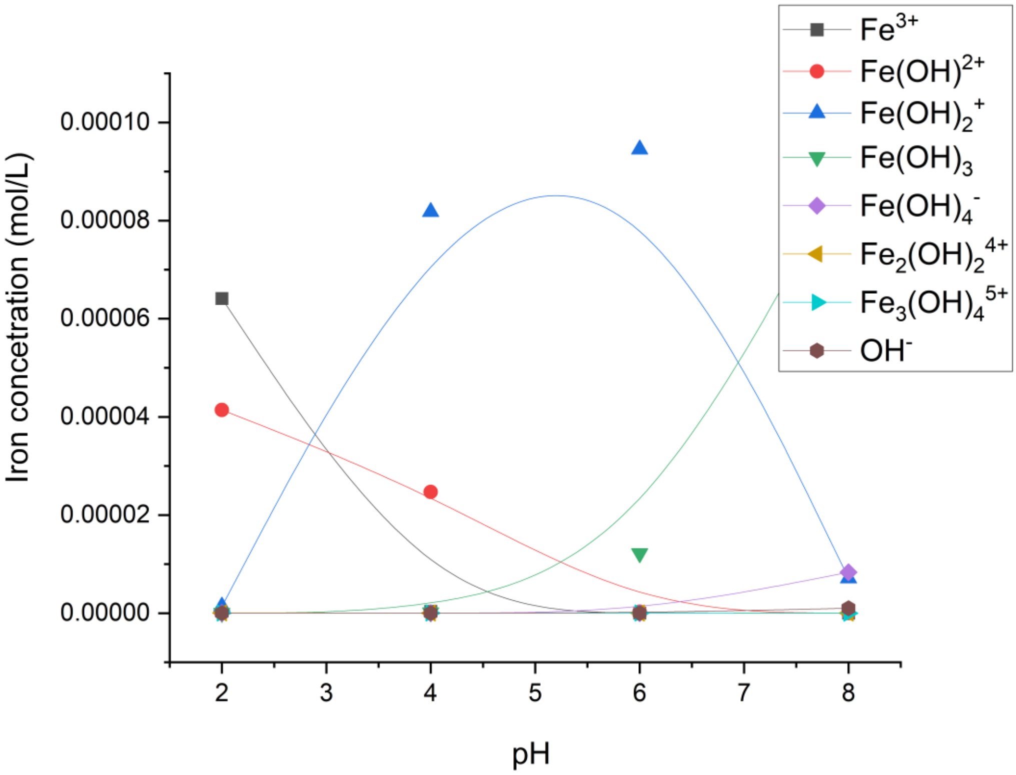

Figure S12. Distribution of Fe (III) species across different pH levels obtained with program ChemEQL V3.2.

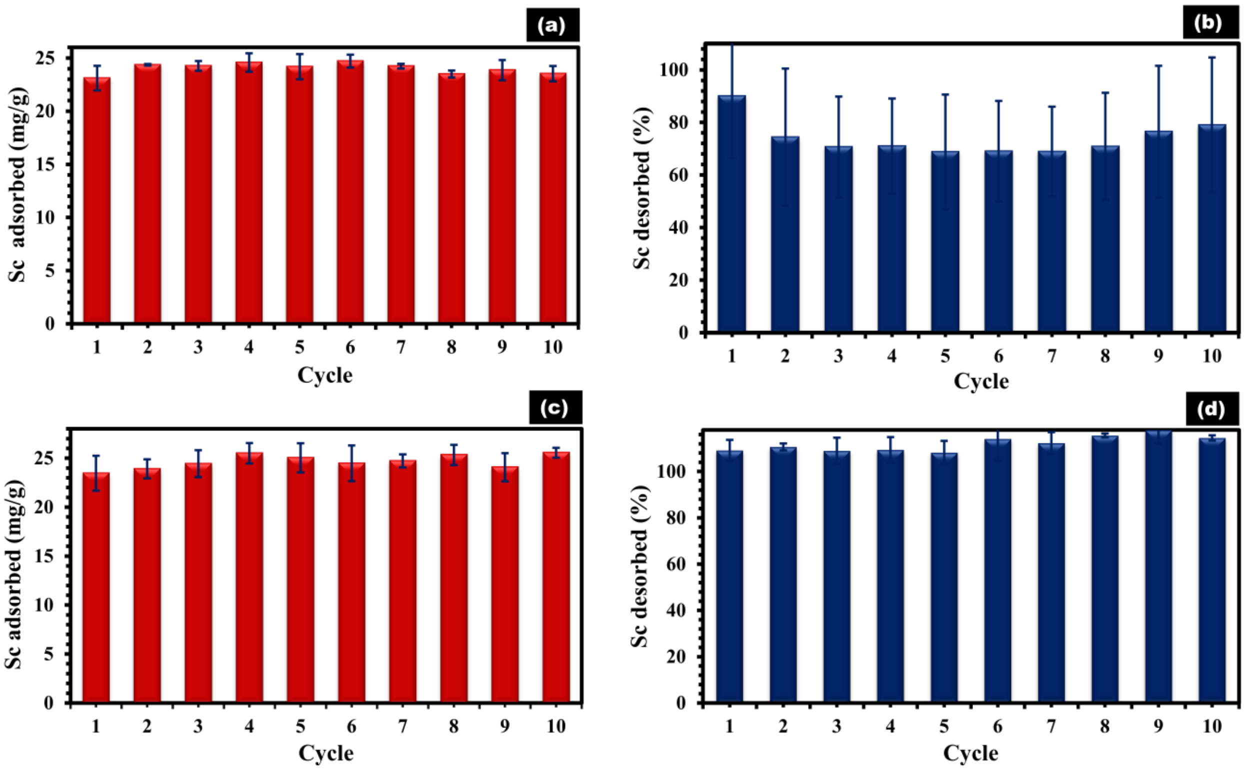

Figure S13. Re-use test of SiO_2/PMDA studied with Sc ions showing adsorption (a) and desorption (b) profiles over 10 cycles; re-use test for SBA-15 studied with Sc ions showing adsorption (c) and desorption (d) profiles over 10 cycles. Tests were performed at room temperature and at pH=4.

References

[1] C. Geibel, J. Theiner, M. Wolter, M. Kramer, W. Lindner, M. Laemmerhofer, Controllable organosilane monolayer density of surface bonding using silatranes for thiol functionalization of silica particles for liquid chromatography and validation of microanalytical method for elemental composition determination, *J. Chromatogr. A* **2021**, *1653*, 462418.

[2] R. Guillet-Nicolas, F. Bérubé, M. Thommes, M.T. Janicke, F. Kleitz, Selectively Tuned Pore Condensation and Hysteresis Behavior in Mesoporous SBA-15 Silica: Correlating Material Synthesis to Advanced Gas Adsorption Analysis, *J. Phys. Chem. C* **2017**, *121*, 24505-24526.

[3] C. Iriarte-Mesa, M. Pretzler, C. von Baeckmann, H. Kählig, R. Krachler, A. Rompel, F. Kleitz, Immobilization of Agaricus bisporus Polyphenol Oxidase 4 on mesoporous silica: Towards mimicking key enzymatic processes in peat soils, *J. Colloid Interface Sci.* **2023**, *646*, 413-425.
